# The human gut microbiota in IBD, characterizing hubs, the core microbiota and terminal nodes: a network-based approach

**DOI:** 10.1186/s12866-025-04106-0

**Published:** 2025-06-26

**Authors:** Theresa Geese, Corinna Bang, Andre Franke, Wolfgang Lieb, Astrid Dempfle

**Affiliations:** 1 Institute of Medical Informatics and Statistics, Kiel University and University Hospital Schleswig-Holstein, Brunswiker Straße 10, 24105 Kiel, Germany; 2 Institute of Clinical Molecular Biology, Kiel University, Kiel, Germany; 3 Institute of Epidemiology, Kiel University, Kiel, Germany

**Keywords:** Inflammatory bowel disease, Gut microbiota, Hub and terminal nodes, Graphlets, Core microbiota, Network analysis, Centrality measures

## Abstract

**Background:**

Dysbiosis, an imbalance in the bacterial composition of the human gut microbiota, is linked to inflammatory bowel disease (IBD). Advances in biological techniques have generated vast microbiota datasets, presenting both opportunities and challenges for clinical research in that field. Network theory offers powerful tools to analyze these complex datasets.

**Methods:**

Utilizing genetically unrelated individuals from the Kiel IBD-KC cohort, we compared network properties of the gut microbiota between patients with inflammatory bowel disease (IBD, *n* = 522) and healthy controls (*n* = 365), and between Crohn's disease (CD, *n* = 230) and Ulcerative Colitis (UC, *n* = 280). Correlation-based microbial networks were constructed, with genera as nodes and significant pairwise correlations as edges. We used centrality measures to identify key microbial constituents, called hubs, and suggest a network-based definition for a core microbiota. Using Graphlet theoretical approaches, we analyzed network topology and individual node roles.

**Results:**

Global network properties differed between cases and controls, with controls showing a potentially more robust network structure characterized by e.g., a greater number of components and a lower edge density. Local network properties varied across all groups. For cases and both UC and CD, *Faecalibacterium* and *Veillonella*, and for unaffected controls *Bacteroides*, *Blautia*, *Clostridium XIVa*, and *Clostridium XVIII* emerged as unique hubs in the respective networks. Graphlet analysis revealed significant differences in terminal node orbits among all groups. Four genera which act as hubs in one state, were found to be terminal nodes in the opposite disease state: *Bacteroides*, *Clostridium XIVa*, *Faecalibacterium*, and *Subdoligranulum*. Comparing our network-based core microbiota definition with a conventional one showed an overlap in approximately half of the core taxa, while core taxa identified through our new definition maintained high abundance.

**Conclusion:**

The network-based approach complements previous investigations of alteration of the human gut microbiota in IBD by offering a different perspective that extends beyond a focus solely on highly abundant taxa. Future studies should further investigate functional roles of hubs and terminal nodes as potential targets for interventions and preventions. Additionally, the advantages of the newly proposed network-based core microbiota definition, should be investigated more systematically.

**Supplementary Information:**

The online version contains supplementary material available at 10.1186/s12866-025-04106-0.

## Introduction

Inflammatory Bowel Diseases (IBD), encompassing mainly Crohn's disease (CD) [1] and Ulcerative Colitis (UC) [2] are chronic inflammatory disorders that are associated with significant morbidity. Despite overlapping symptoms, CD and UC exhibit distinct characteristics. UC primarily affects the colon and rectum, in contrast CD most commonly involves the terminal ileum and large intestine [3]. In CD inflammation typically affects all layers, whereas in UC it is confined to the mucosa [4]. While both conditions impact patient's quality of life, CD is often perceived as more severe by patients [5]. The global prevalence almost doubled between 1990 (3.7 million) and 2017 (6.8 million) [6]. IBD is a multifactorial disease and part of its development and progression can be linked to a dysbiosis of the gut microbiome, an alteration of the microbiome composition [7]. Other risk factors include genetic predisposition [8], smoking, environmental factors [9], and dietary factors [10]. The gut microbiome is a complex and dynamic ecosystem consisting of trillions of microorganisms that interact with each other and with the host in complex ways. It also plays a crucial role in maintaining host health, including regulating metabolism, immune function, and even brain function [11]. Identifying important components of the gut microbiota in IBD is of great interest, as it may unveil potential avenues for preventive measures and therapeutic interventions. Studies have shown that the gut microbiome in IBD patients differs significantly from that of healthy individuals, with notable reductions in microbial diversity and richness [12, 13]. These changes extend to the overall composition, where some bacterial taxa are observed to be either depleted or enriched in IBD patients [7]. These compositional and functional alterations in the microbiome are not only linked to disease mechanisms, but also hold promise for clinical applications. In particular, the gut microbiome represents a promising non-invasive diagnostic tool for IBD [14], as well as a therapeutic target [15].

The collection of vast microbiome data sets in recent years presents a unique opportunity for comprehending the complex microbial communities within the human body. But it also presents a significant challenge for researchers to handle and analyze such complex data. Network theory has emerged as a potent tool to better understand the complexities of biological systems, for example when analyzing protein–protein interaction networks [16]. Network theory can complement other statistical methods by providing researchers with the means to investigate the relationships between individual components within a system and their interaction as a whole. Two types of networks can be distinguished: data-driven networks, which are statistical networks based on data, and knowledge-driven networks, which utilize prior biological knowledge. For instance, co-occurrence and correlation-based networks represent the former, whereas metabolic networks constructed using pathways exemplify the latter.

When constructing microbial correlation-based networks, taxa serve as nodes which are connected through edges based on measures of association. Edges exist between pairs of taxa that exhibit significant co-occurrence patterns across samples. For instance, Faust et al. [9] employed network-based methods to analyze the microbial communities and found that such networks can reveal complex interactions that are not immediately obvious through traditional methods. However, there are several difficulties in construction and interpretation, such as data normalization. A study by Abbas et al. [17] highlighted how microbial network-based feature selection could be used for identifying IBD biomarkers, particularly in distinguishing disease phenotypes from healthy controls. Their approach integrated network-wide and node-level measures, such as centrality and resilience, to refine biomarker discovery. Identifying which are the most important taxa remains a challenge; however, it is of great interest in the study of IBD to understand their function. A set of taxa that is characteristic for a host or environment oftentimes is called the core, typically defined based on thresholds of abundance and prevalence, see for example [18]. However, abundance and prevalence alone do not necessarily denote importance, and establishing cut-off values is arbitrary. For instance, thresholds for relative abundance range from 0.001 to 4.5%, while prevalence cut-offs range from 50 to 100% [18]. These criteria might overlook other crucial factors, such as the role of specific taxa in maintaining overall community structure [19], potentially leading to an incomplete understanding of the core microbiota's composition and stability. Furthermore, with differences in clinical factors and the microbiota observed across subtypes of IBD, more analyses to unravel the nuanced differences in CD and UC are needed [20]. A different method to characterize important nodes are centrality concepts. Since centrality is inherently a rank-based metric, it does not require the application of an explicit threshold. Thresholds are only necessary when identifying specific subsets, to define and distinguish nodes of interest within the network. The concept of centrality in co-occurrence networks as a proxy for importance has been supported in various microbiome studies, like protein–protein networks [21], co-occurrence networks for soil microbiota [22] and in the microbial community [23]. Nodes with high centrality often represent"keystone"taxa that can exert significant influence over microbial community structure and stability [24].

As outlined above, network analysis has been applied in several studies investigating the human gut microbiome in the context of IBD. However, defining important nodes or taxa remains challenging and even network-based approaches often rely on abundance or prevalence, assuming that highly abundant taxa are also the most influential. Moreover, nuanced differences between CD and UC have not yet been investigated very much within network-based frameworks. Additionally, existing studies report heterogeneous findings regarding differences in local and global network properties. Most network analyses focus on describing these properties but often cannot provide detailed insights into the role of individual nodes, due to limitations inherent in the network construction process.

In this study, we employed data from the IBD Kindred cohort (IBD-KC) [25], comprising a collective sample size of over 887 subjects from Northern Germany, including 522 IBD patients (in the following referred to as cases) and 365 healthy controls (in the following referred to as controls). Our goal was to employ network-based approaches to characterize the gut microbiota in both health and disease. We constructed microbiota networks, using 16S rRNA gene abundance data separately for cases and controls, as well as for CD and UC patients. These networks were analyzed to assess global and local properties, providing insights into structural differences between healthy and dysbiotic microbiomes. By assessing the centrality of nodes, we described the importance of genera and identified hubs, genera which could potentially be serving as critical players in the difference between a healthy and a dysbiotic state of the microbiota. Furthermore we used those hubs to provide a different definition for the core microbiota. Additionally, we utilized graphlet theoretical approaches [26, 27] to analyze the network's topology and discern the roles of individual nodes. This enabled us to unravel patterns of connectivity and uncover distinct patterns within the microbiota network.

## Methods

### Study design and population

The Kindred cohort (IBD-KC) is a family-based IBD cohort, where families were recruited based on at least one patient with IBD. Briefly, the IBD-KC is a prospective study initiated in 2013 in Kiel, Germany, and ongoing since then, currently including 1715 study participants. We used the cohort as a case–control study design, including index patients (with IBD) as cases and unaffected family members as controls. Recruitment of IBD patients was carried out through treating physicians, clinics, study flyers, letters, and information disseminated by patient organizations like the German Crohn's Disease/Ulcerative Colitis Association. Biomaterial examples including stool are available, see supplement for details of gut microbiome data generation and preprocessing. Questionnaires were filled out in a self-reported manner and for cases in addition by their treating physician, from which IBD diagnoses were curated to be grouped into the correct subtype.

Using only genetically unrelated individuals (R package kinship2 version 1.9.6.1, function pedigree.unrelated) as cases and controls, at baseline, a total of 887 participants were used for the current analysis, including 365 healthy controls and 522 cases (230 with ulcerative colitis (UC), 280 with Crohn's disease (CD), and 12 with undefined colitis). Despite only including genetically unrelated individuals, meaning any two individuals are unrelated irrespective of case or control status, similar environments might affect the analysis.

### Network-based analysis

To gain a comprehensive descriptive overview, we initiated the analysis by following traditional steps to confirm expected differences in established measures between cases and controls [28]. Here, we are mainly interested in the feature diversity within individual samples and comparing them across groups (cases and controls, and CD and UC), therefore we focus on alpha diversity. The Chao1 index (R package fossil version 0.4.0) is a measure of richness, whereas the Shannon index (diversity function from the R package vegan version 2.6–4) is a measure of evenness.

#### Data preparation

To construct microbial correlation-based networks, 16S rRNA gene microbial abundance data was used. The initial step in preparing the data, splitted into groups, involves filtering out a specific set of genera and samples. Filtering steps help reduce the complexity of data and technical variability, and create more reproducible results [29, 30]. Genera are kept if: (i) the number of reads averaged over all samples is at least 0.001% of the total number of reads and (ii) they are observed in at least 1% of the samples. We consider these cutoffs robust, as supported by sensitivity analyses in other studies examining the impact of filtering steps, such as e.g. [31]. Samples are kept if the total number of reads after quality control is at least 10,000. These filtering steps lead to a reduction from 548 to 87 genera and 11 fewer controls (354 from 365), and three fewer cases (519 from 522). Applying the same filtering steps separately for the networks for CD and UC leads to a reduction from 548 to 86 genera, and one fewer sample for UC (229 from 230) and two fewer samples for CD (278 from 280).

To address the compositional nature of the data and allow the use of correlation-based methods, we applied a centered log-ratio (CLR) transformation. Prior to the CLR transformation, zero values were handled using a Bayesian approach (function cmultRepl from the R package zCompositions version 1.5.0–3), which replaces zeros with small positive values based on the data distribution. As association measure between genera we used Pearson correlation after log-ratio transformation, as implemented in SparCC [32]. This has been proposed and is widely used for sparse, compositional data such as microbiome abundances.

#### Network construction

Using the NetCoMi R package [33], we constructed microbial correlation-based networks separately for controls, for cases, for CD patients, and for UC patients. Microbial genera serve as nodes and edges represent the Pearson correlation coefficient between pairs of genera, computed using the SparCC algorithm [32]. Two nodes are connected through an edge, if the correlation between those nodes significantly differs from zero.The resulting network reveals patterns among different microbial genera. We retained statistically significant edges based on a one-sample Student’s t-test as a sparsification method. Taking into account multiple testing, *p*-values are adjusted using the local false discovery rate correction, with a family-wise significance level set at 0.05. To identify clusters within a microbial network, groups of highly connected nodes, the cluster-fast-greedy algorithm is used [34]. For visualization purposes, the"spring layout"in the R package is employed with the netPlot function. This approach should be distinguished from differential network analysis, where the abundance of a genus in one group is subtracted from its abundance in another group, resulting in a single combined network without group-specific partitions. This can lead to false differential edges caused by the change of conditional variances. The need for rigorous statistical methods requires more complex models to ensure the robustness of the results [35].

#### Network analysis

The resulting networks are further analyzed based on global and local network characteristics. The following global network properties were calculated: (1) Number of components [36]: subnetworks where any two nodes are connected by a path, each single unconnected node is a component too, as connected to itself via the trivial path, (2) Clustering coefficient [37]: indicates how likely it is for neighboring nodes of a particular node to be connected to each other, a high clustering coefficient indicates a network where nodes tend to form clusters, (3) Modularity [34]: is a measure to evaluate the quality of a division of a network into clusters, measuring the number of within cluster edges, relative to a null model of a random network, (4) Positive edge percentage [38]: indicates the percentage of edges with positive estimated correlation of the total number of edges, (5) Edge density [39]: the proportion of present edges relative to the number of possible edges in the network, (6) Edge number: considering that edge density is a relative measure of network connectivity, the product of edge density and the number of components, referred to as edge number, provides a more comprehensive assessment of network complexity, and (7) Natural connectivity [40]: robustness measure, corresponding to the average eigenvalue of the adjacency matrix. Other measures were only calculated for the largest connected component (LCC): (8) Relative LCC size [36]: calculated as number of nodes in the LCC divided by number of nodes in the whole network, (9) Average dissimilarity [41]: dissimilarity is defined as 1- edge weight, where the edge weight is the absolute value of the strength of the correlation coefficient between pairs of nodes, and (10) Average path length [42]: average number of steps along the shortest path for all possible pairs of connected nodes.

#### Local properties/Hubs

In order to describe the importance and influence of nodes within the network we calculated node centrality measures. In this study degree, betweenness, and closeness centrality are used. Normalized versions of betweenness and closeness were calculated, by dividing the centrality measure by n-1, n being the number of nodes in the network. For better comparability, all centrality measures were min/max scaled. Betweenness centrality is defined as the number of shortest paths that pass through a node, indicating the importance of a genus as a bridge, connecting different parts of the microbiota network [43, 44]. Closeness centrality is based on distances, i.e. the lengths of paths to all other genera in the network [45]. If a node is unconnected its closeness centrality is considered zero or undefined. Degree centrality, simply the number of edges of a node, quantifies the number of connections that a genus has with other genera in the network, indicating that node's overall importance in the network [46]. An important genus in terms of node degree will have many neighbors.

Hub nodes are defined as nodes with the highest centrality for all selected measures. Specifically, nodes with each centrality value above the 90th percentile are defined as hubs. We chose to characterize important nodes as hubs in the microbiome network rather than relying solely on abundance and prevalence, as hubs provide a more comprehensive view of their importance. For example, Fisher et al. [47] identified *Bacteroides* as a"keystone species"in microbial communities, despite its relatively low median abundance. While abundance measures the commonness of a taxon, centrality metrics, such as degree and closeness, are more indicative of a taxon’s influence on structure and stability [48, 49]. A study by Berry et al. [23] has demonstrated that utilizing high degree and closeness centrality, can identify keystone taxa with 85% accuracy.

Additionally, the Jaccard index, as proposed by [50], was computed for each centrality measure, expressing for each centrality measure how similar the sets of most central nodes (above the 0.90 quantile) are among two networks.

#### Core microbiota

In our study, we examined the core microbiota separately for cases and controls, as well as for CD and UC, at the genus level. Oftentimes the definition of the core microbiota is based on prevalence metrics, alongside relative and/or absolute abundance considerations, with typical thresholds being 50/100 for prevalence and 0.1/100 for abundance [18]. We suggest a different definition of the core microbiota making use of the concept of hub nodes, by considering all identified hub genera as core members. To enhance comparability between both definitions, we lowered the percentile in the definition of hubs, until the same number of genera is achieved as in the used abundance/prevalence core definition. We investigated the overlap in terms of core microbiota members between cases and controls, as well as between CD and UC. To further compare group wise, we calculated for all genera in the core and with both definitions separately: (i) the average prevalence: average percentage of core genera present per sample, (ii) the cumulative total abundance: abundances of core genera summed for genera and averaged over samples, and (iii) the averaged value of all centrality measures. For these analysis steps we created phyloseq objects [51] and used the R package microbiome [52] and its functions “transform” and “core_members”.

#### Network comparison/Differential network analysis

In the last step, two constructed networks (controls vs. cases and CD vs. UC) can be compared, regarding local and global network properties.

Permutation tests are used to compare local and global network properties between two groups. The null-hypothesis is defined as ${H}_{0 }: {nProps}_{1}= {nProps}_{2}$, where ${nProps}_{1}$ and ${nProps}_{2}$ denote the local/global network property in group 1 and 2 respectively. To obtain sampling distributions of the differences under the null hypothesis, we employ standard nonparametric permutation procedures. This involves randomly reassigning group labels to the samples while maintaining the original group sizes. Similar steps are followed to assess significant differences in the global network measures for both groups. To assess the difference between the two sets of most central nodes (hubs) the Jaccard index is used, ranging from 0 (no nodes in common) to 1 (equal sets). To test if that index is different from a randomly expected index, a permutation-based approach (1000 permutations) following Real and Vargas [50] is applied.

#### Graphlets

**Fig. 1:**
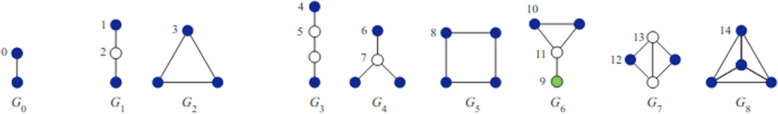
Graphlets up to four nodes. Graphlets with k = 2 to k = 4 nodes. Node colors correspond to orbit types within each graphlet, labeling refers to orbits. Adapted from [26]

By analyzing the distribution of graphlets and orbits within a network, one can identify recurring patterns of connectivity that are not immediately apparent from an examination of the entire network. Graphlets are induced sub-graphs of *k* connected nodes from the complete graph [26, 53], see Fig. 1. Orbits contain nodes, which when swapped result in an automorphism of the same graphlet. Grouping nodes into orbits allows one to analyze the individual role of nodes better, as orbits represent different local network structures. Regarding $k=4$ nodes, there are 11 non-redundant orbits: Orbit 0 represents the node degree, while orbits 2, 5, and 7 represent nodes within a chain, orbits 8, 10, and 11 represent nodes in a cycle, and orbits 6, 9, 4, and 1 represent terminal nodes. To compute the graphlet correlation matrix (GCM) [54], we followed a series of steps. First, we computed a matrix containing for each node the orbit’s degree, that is, the number of times the node is present in each orbit. The columns of this matrix are called the graphlet degree distribution and rows are called graphlet degree vectors, allowing for the analysis of the roles of individual nodes. By computing Spearman's correlation coefficient between all pairs of columns from the above described matrix, one ends up with the GCM. Here, we used the Spearman correlation instead of the Pearson correlation. Being rank-based, Spearman correlation can detect monotonic but non-linear relationships, making it well-suited for identifying broader patterns of association [53]. To compare GCMs between two groups, we created a new GCM by subtracting the entries from the upper triangles of each GCM. Subsequently, we conducted a Fisher’s z-test to assess the significance of the absolute differences between graphlet correlations in this new GCM [26, 53]. Only orbits up to $k=4$ nodes are taken into account, and orbit counts are calculated using the function count4 from the orca package in R [26]. Additionally, a row with pseudo-counts of 1 is added, to deal with the problem of missing values that would occur for unobserved orbits.

By comparing two networks and their GCMs, we chose orbits of interest, based on the discrepancy in pairwise similarity of subgraph patterns. Specifically, we focus on orbits where the similarity between corresponding entries in the two distinct GCMs significantly varies in terms of Spearman correlation coefficient values. We propose that nodes with similar local network structures, predominantly occurring in the same orbits, may exhibit similar metabolic functions. This idea aligns with findings in the literature [55, 56], showing an association between co-occurrence and metabolic dependence. Therefore, we map genera back to orbits of interest to gain biological insights. To map genera back, we use the graphlet degree distribution, arranging the rows with orbits of interest by genus occurrence within each selected orbit. By selecting the top 25 genera for each orbit, we identify common genera across all selected orbits. The choice of the top 25 genera per orbit was a practical one, balancing focus on prominent genera with manageable analysis size. These shared genera serve as focal points for comparative analysis between groups, such as cases versus controls, as well as between CD and UC.

## Results

### Characterization of IBD-KC cohort

Clinical and gut microbiota-specific data of IBD cases and healthy controls from the IBD-KC cohort is given in Table 1. There are significant differences in all of those variables between cases and controls, suggesting to adjust for those variables. However, network analysis does not easily allow for the inclusion of covariates [57]. Here, stratification would be the only feasible approach, which would severely reduce the sample size. Sample size is critical for network analysis [58], particularly when conducting permutation tests. Given the importance of maintaining an adequate sample size, we chose not to pursue stratification in this study.

**Table 1:** Characterization of the IBD-KC cohort

	**IBD-KC**		
	**IBD cases**	**non IBD controls**	***p***_***IBD***_^***a***^
Subjects, n	522	365	-
Female sex, n (%)	342 (65.52)	193 (52.88)	8.8 × 10^–3^
Age, years	56 (44, 64)	60 [49, 70]	< 10^–5^
BMI, kg/m^2^	24.01 [21.72, 26.81]	25.08 [22.48, 27.99]	1.15 × 10^–4^
Current Smoker, n(%)	54 (10.34)	34 (17.62)	9.3 × 10^–3^
*Gut microbiota*			
Shannon index (evenness)	2.19 [1.89, 2.40]	2.30 [2.11; 2.46]	< 10^–5^
Chao1 index (richness)	43 [35, 49.75]	46 [41, 52]	< 10^–5^

Data is displayed as median [Interquartile Range], or count n (percentage)

*IBD* Inflammatory bowel disease; *BMI* Body Mass Index

^a^Chi^2^-test for categorical variables, Mann–Whitney test for continuous variables

Preceding the network-based analysis of the human gut microbiota, we analyzed some characteristics of the human gut microbiota in the context of IBD and its subtypes.

**Fig. 2:**
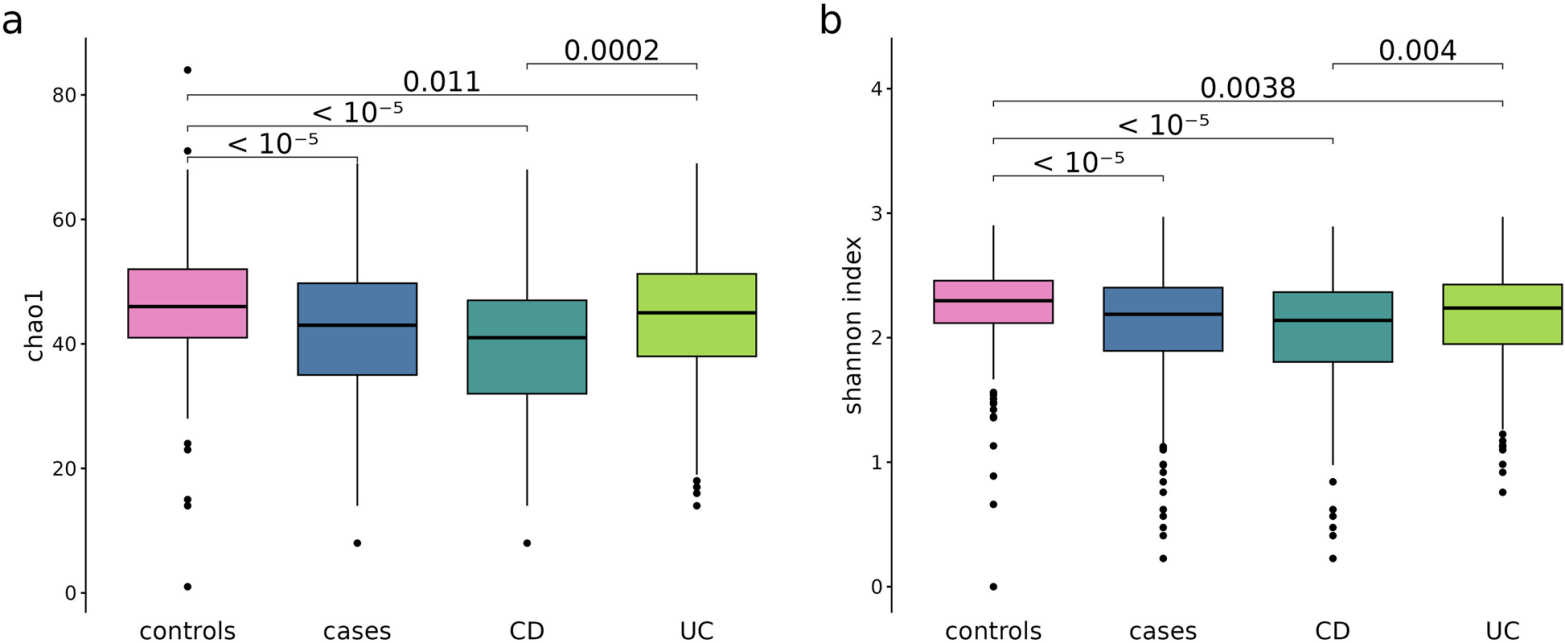
Alpha diversity expressed through the Shannon index (**a**) and Chao1 index (**b**), colors depict the different groups. Both indices show higher diversity in the control group, and CD patients having the lowest diversity. Significance is tested using the Mann–Whitney test

As expected, the analysis demonstrated significantly reduced microbial richness (Chao1 index, Fig. 2a) in individuals with IBD compared to unaffected controls, with CD exhibiting significantly lower values than UC. Additionally, IBD cases displayed significantly decreased microbial evenness (Shannon index, Fig. 2b), particularly evident in CD compared to UC again.

### Global properties

Following the network construction, we analyzed global properties of the networks and their largest connected component (LCC), and compared them between cases and unaffected controls, as well as between CD and UC patients, see Table 2. Furthermore, we compared CD to controls and UC to controls separately, see Supplementary table S T1.

**Table 2:** Global network properties

**Global Network properties**	**Controls**	**Cases**	*P*_controls/cases_	**CD**	**UC**	*P*_CD/UC_
Number of components	10	2	0.002	3	5	0.65
Clustering coefficient	0.45	0.62	0.001	0.55	0.49	0.28
Modularity	0.34	0.13	0.001	0.15	0.19	0.52
Positive edge percentage	68.00	50.15	0.001	53.99	57.72	0.34
Edge density	0.09	0.36	0.001	0.23	0.15	0.17
Natural connectivity	0.023	0.10	0.001	0.073	0.043	0.13
Edge number	0.9	0.72	n.a.^c^	0.69	0.75	n.a.^c^
Largest connected component (LCC)						
Relative LCC size	0.90	0.99	0.002	0.98	0.95	0.65
Average dissimilarity^a^	0.96	0.89	0.001	0.92	0.95	0.14
Average path length^b^	1.82	1.24	0.001	1.38	1.54	0.18
Clustering coefficient	0.45	0.62	0.001	0.55	0.50	0.28
Modularity	0.34	0.13	0.001	0.15	0.19	0.52

Comparison of global network properties between controls and cases, as well as between CD and UC patients. *P*-value obtained through a permutation test (1000 permutations). ^a^Dissimilarity = 1—edge weight, ^b^Path length = Units with average dissimilarity, ^c^*p*-value not available, due to specific definition of edge number

**Fig. 3:**
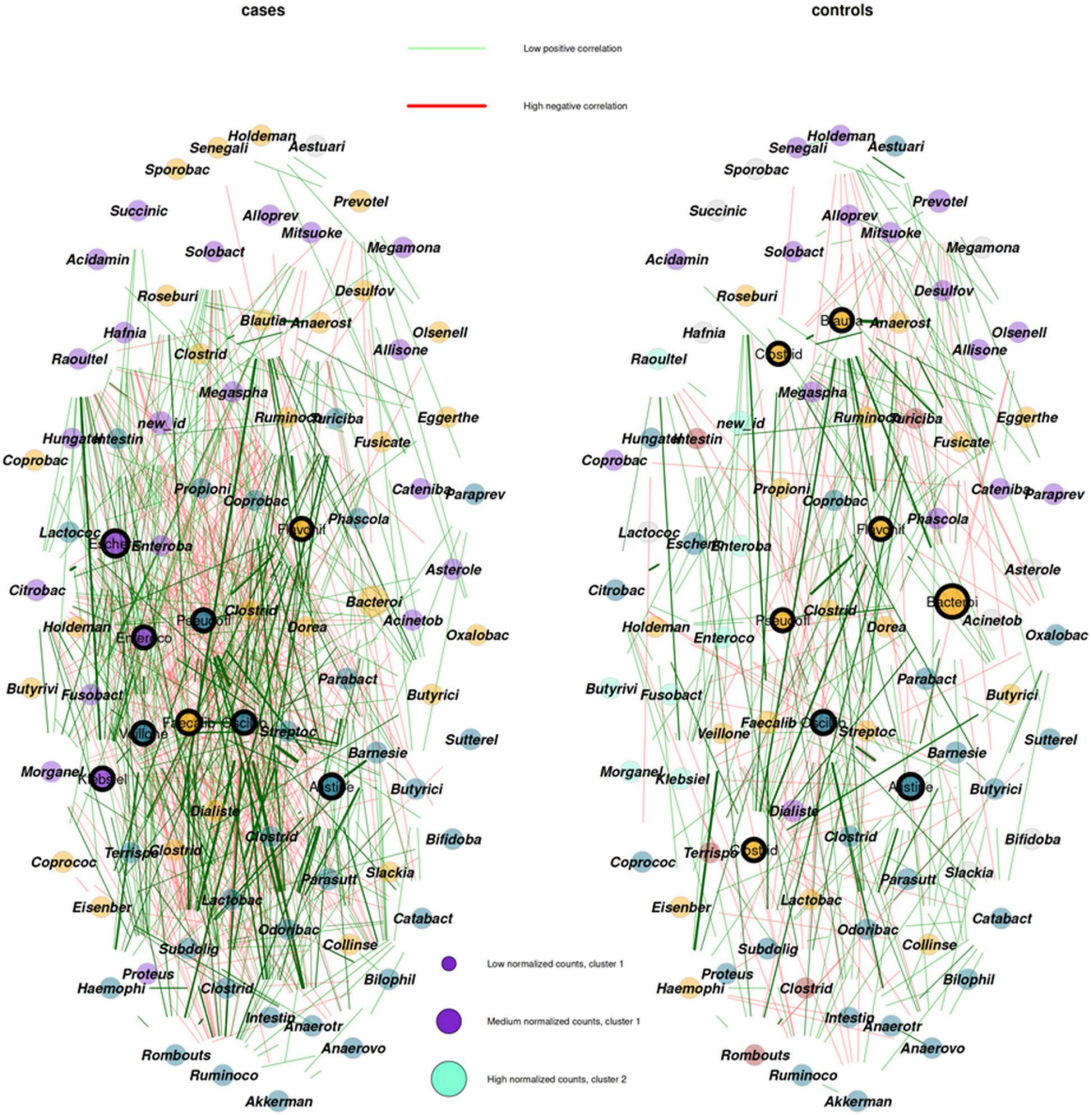
Node color depicts the cluster, node size is scaled according to the sum of normalized counts, hubs are highlighted by a black circle, and for clarity only edges corresponding to an absolute association > = 0.15 are plotted and labels of nodes are shortened. The color and thickness of the edges indicate the direction (red for negative, green for positive) and strength of the Pearson correlation coefficient. Same layout is used for both groups and gray nodes depict genera that are not connected and/or only present in the other group

Cases and controls demonstrated differences in both global network properties, as well as characteristics specific to the largest connected component (LCC). Notably, the number of components (already a single unconnected node counts as a component) differed significantly between cases and controls, as well as the number of clusters (see Fig. 3). The clustering coefficient and natural connectivity were significantly higher in cases compared to controls. Controls exhibited significantly higher modularity and displayed a higher percentage of positive edges, indicating more microbial genera correlated within their networks. Controls also exhibited lower edge density compared to cases, suggesting that while the overall number of connections may be lower, the connectivity within individual components is higher. The same can be seen regarding the higher edge number observed in controls, which suggests a greater level of connectivity within their microbial networks. This phenomenon is visually depicted in Fig. 3 and explains the significantly higher number of components observed in the network of controls. The size of the LCC was notably larger in cases, suggesting a more consolidated and interconnected microbial community in individuals with IBD. This observation, coupled with the higher clustering coefficient observed in cases and higher modularity in controls, further emphasizes the contrasting organization of microbial networks between cases and unaffected controls. Additionally, the higher average path length and dissimilarity within the LCC of healthy individuals indicate a greater degree of network compactness and strength.

In contrast, both CD and UC groups displayed similarity, with no significant differences detected in global network properties. This similarity is also visually represented in Figure S1.

Additionally, when investigating differences in global network properties between UC and controls, and CD and controls, we observed interesting results. No significant differences were found between UC and controls, but most properties showed significant differences between CD and controls. A similar pattern was observed for microbial richness and evenness (see Fig. 2), where CD exhibited significantly lower values than UC, highlighting the distinct characteristics of the CD group.

The global property values for cases do not fall within the range observed for CD and UC but are notably closer to them compared to the values for controls. This discrepancy may arise from variations in the network construction process, which relies on different sub datasets, as well as the number of components identified in each group.

### Local properties

In the analysis of local networks, the first step is selecting appropriate centrality measures to identify hub nodes. Next, hub nodes are used to propose an alternative definition for the core microbiota. Finally, the role of individual nodes is examined through graphlet analysis and GCM’S.

### Identification of Hubs

We compared hubs of networks constructed using microbial abundance data from cases and unaffected controls, as well as from CD and UC patients. By utilizing three node centrality concepts, namely betweenness, closeness, and degree centrality, we were able to define hubs (definition see network-based analysis).

The Jaccard index was utilized to assess the similarity between the sets of most central nodes (above the 0.90 quantile) for each centrality measure, across different networks, see Table 3. Comparisons between cases and controls revealed a very low level of similarity, with a Jaccard index around 0.3. Comparisons between CD and UC showcased a slightly higher, but still non-significant level of similarity. For example, when using closeness centrality to compute the Jaccard index, the index is right in the middle of the interval (0.50).

**Table 3:** Comparison of local network properties

**Comparison**	**Controls vs Cases**	*P*_controls/cases_	**CD vs UC**	*P*_CD/UC_
Jaccard index				
Degree	0.29	0.48	0.38	0.45
Betweenness centrality	0.29	0.48	0.38	0.45
Closeness centrality	0.29	0.48	0.50	0.18

Similarity between the set of most central nodes accessed through the Jaccard index. Comparing between controls and cases, as well as between CD and UC patients. Most central set of nodes defined via one of the three centrality concepts: degree, betweenness, or closeness centrality. *P*-value obtained through permutation test (1000 permutations)

**Fig. 4:**
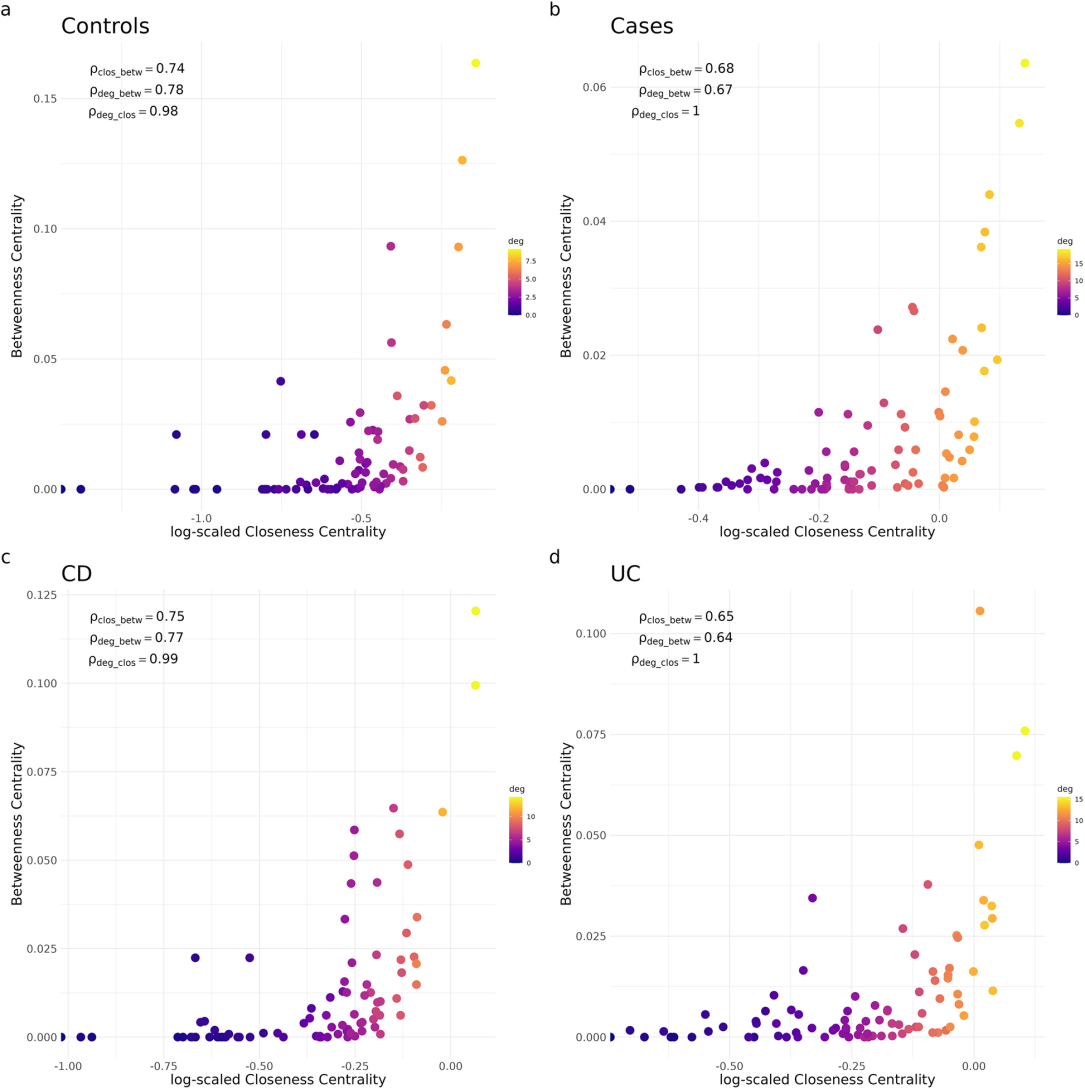
Closeness against betweenness centrality measures for all genera of networks constructed for cases (**a**) and controls (**b**), CD (**c**) and UC (**d**), color scale represents degree value. For better visibility the x-axis is log-scaled. Additionally, each subplot displays the Spearman correlation coefficients for the associations between closeness and betweenness (clos_betw), degree and betweenness (deg_betw), and degree and closeness (deg_clos)

Based on similar Jaccard values for all three centrality measures, we investigated further, how the three centrality concepts are related with each other. Literature varies on the if and to what extent centrality concepts are correlated [23, 59]. To address this, we performed an analysis to explore the nuances of these centrality metrics within the context of our specific microbiome networks. This approach allowed us to ensure accurate interpretation of the metrics and to provide a clear rationale for selecting the centrality measures used in our analysis. Figure 4 displays min/max normalized betweenness, closeness and degree of individual genera in controls (a), cases (b), CD (c), and UC (d) patients.

The results demonstrated a consistent trend: closeness centrality is moderately correlated with betweenness centrality, as reflected by Spearman correlation coefficients around ρ ≈ 0.7. Degree showed a particularly strong correlation with closeness (ρ = 0.98–1), and a mild to moderate correlation with betweenness (ρ = 0.65–0.75). These findings align with the conceptual distinctions between the measures. While degree and closeness are both indicators of local node connectivity, betweenness captures the extent to which a node lies on global paths within the network. Hence, it is crucial to examine all three centrality measures when identifying hubs.

**Fig. 5:**
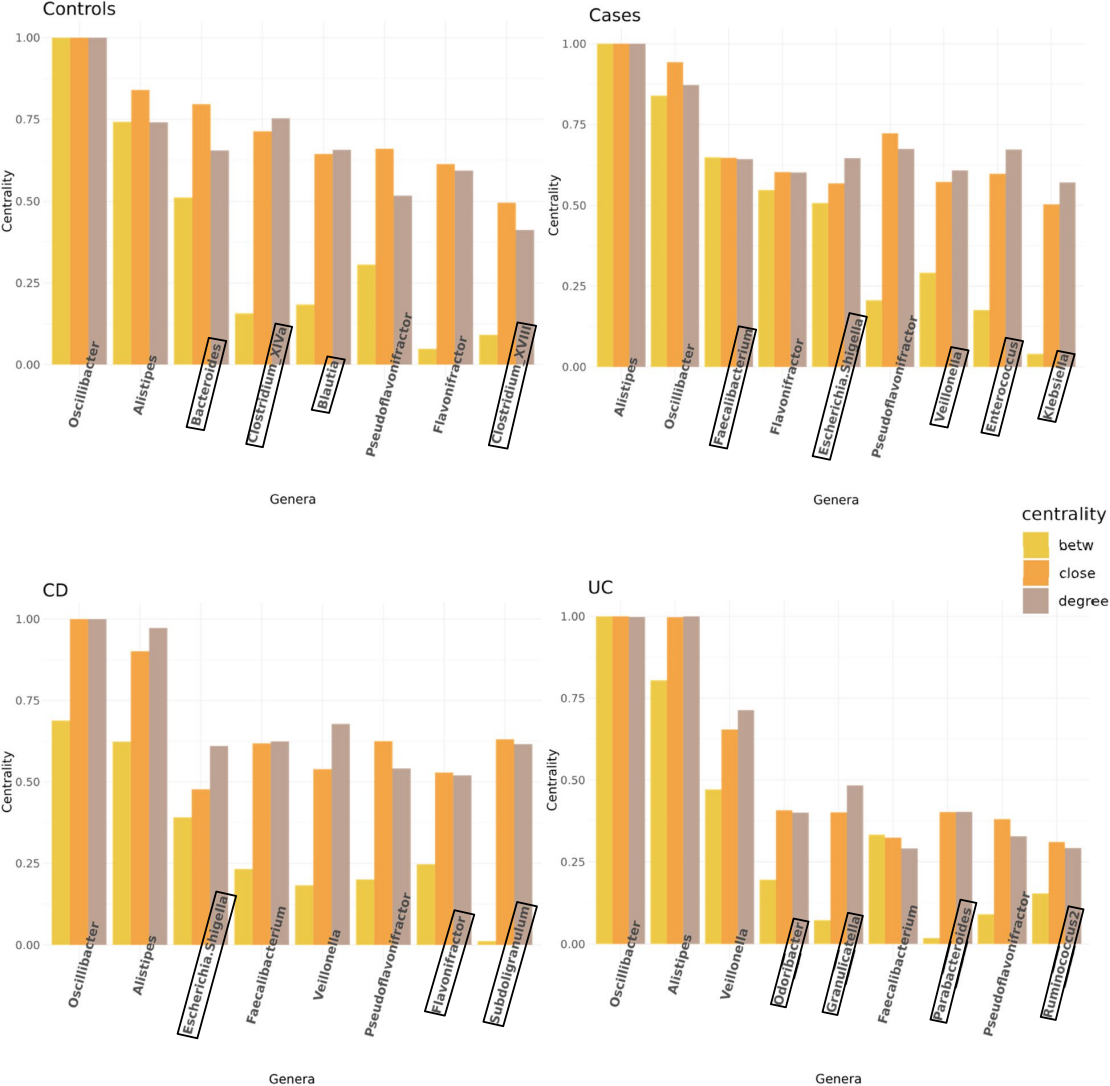
Barplots depict min/max normalized centrality values for betweenness (yellow), closeness (orange), and degree (brown) for hubs from controls, cases, CD, and UC patients. Boxes present genera present in controls but not cases and vice versa, and genera present in CD but not UC and vice versa

Based on the previous analysis steps, hubs, defined as genera in the network for which each of the three centrality measures is above the 0.9 quantile, were identified. The results are displayed in Fig. 5 and Table 4, stratified for IBD cases, unaffected controls, CD, and UC.

**Table 4:** Hub nodes and their summed centrality values

Hubs	controls	cases	CD	UC
*Alistipes*	**2.56**	**3**	**2.63**	**2.82**
*Bacteroides*	**2.29**	1.4	1.43	1.18
*Blautia*	**1.96**	1.83	1.73	1.54
*Clostridium_XlVa*	**2.02**	1.84	1.5	1.8
*Clostridium_XVIII*	**1.69**	1.25	1.01	1.06
*Enterococcus*	1.04	**2.09**	1.88	1.46
*Escherichia.Shigella*	0.79	**2.37**	**2.21**	1.13
*Faecalibacterium*	1.41	**2.51**	**2.1**	**1.82**
*Flavonifractor*	**1.8**	**2.4**	**2.06**	1.83
*Granulicatella*	NA	NA	1.47	**1.72**
*Klebsiella*	1.55	**1.92**	1.72	1.57
*Odoribacter*	1.49	1.85	1.69	**1.78**
*Oscillibacter*	**3**	**2.8**	**2.72**	**3**
*Parabacteroides*	0.99	1.74	1.4	**1.62**
*Pseudoflavonifractor*	**1.99**	**2.14**	**2.04**	**1.64**
*Ruminococcus2*	1.37	1.99	2.69	**1.66**
*Subdoligranulum*	1.23	1.79	**1.9**	1.43
*Veillonella*	0.94	**2.17**	**2.06**	**2.27**

Hubs for controls, cases, CD, and UC in alphabetical order, and summed (degree + betweenness + closeness) min/max normalized centrality value. Genera in bold indicate that those genera are hubs in the respective group (column). NA indicates that this genus was not present in the group specific network

Comparing cases and controls, 4 of 8 hubs for controls were not hubs in cases, while 5 of 9 hubs for cases were not hubs in controls. Similar hubs in cases and controls are these four genera: *Oscillibacter*, *Alistipes*, *Pseudoflavinofractor*, and *Flavonifractor*. *Oscillibacter* exhibits the highest centrality, followed by *Allistipes* and *Pseudoflavonifractor*.

Similarly, 3 of 8 hubs for CD were not hubs in UC, and 4 out of 9 hubs for UC were not hubs in CD. Similar hubs in CD and UC are these 5 genera: *Alistipes*, *Faecalibacterium*, *Oscillibacter*, *Pseudoflavonifractor*, and *Veillonella*.

Notably, *Oscillibacter*, *Alistipes*, and *Pseudoflavinofractor* are hubs in all subgroups, highlighting the significance of those genera in the networks across conditions. *Faecalibacterium* and *Veillonella* emerge as significant contributors in IBD and both subtypes, but not in controls. In controls, their centrality values are between 1 to 1.5 for *Faecalibacterium* and below 1.0 for *Veillonella* which is notably lower than in the other groups, where their centrality values exceed 2.0. *Bacteroides*, *Blautia*, *Clostridium XVIII* and *Clostridium XIVa* stand out as hubs exclusively in controls, with centrality values between 1.69 and 2.59, and are not hubs in IBD or its subtypes. However, for *Blautia* and *Clostridium XIVa* the centrality values in IBD and its subtypes are still comparable high (1.54 to 1.83 and 1.5 to 1.84), indicating these genera still as important nodes. Notably evident in the subplot for cases (see Fig. 5) is that the genera appear to be arranged in order on their betweenness centrality values. This phenomenon can be attributed to the previously observed moderate correlation between those centrality concepts.

### The core microbiota

We used two distinct definitions for characterizing the core microbiota. The first commonly used one, referred to as definition 1, is based on prevalence (50/100) and abundance (0.1/100). In contrast, we propose an alternative definition, definition 2, wherein core microbiota taxa are defined via network centrality measures similar to the previously defined hub nodes.

**Fig. 6:**
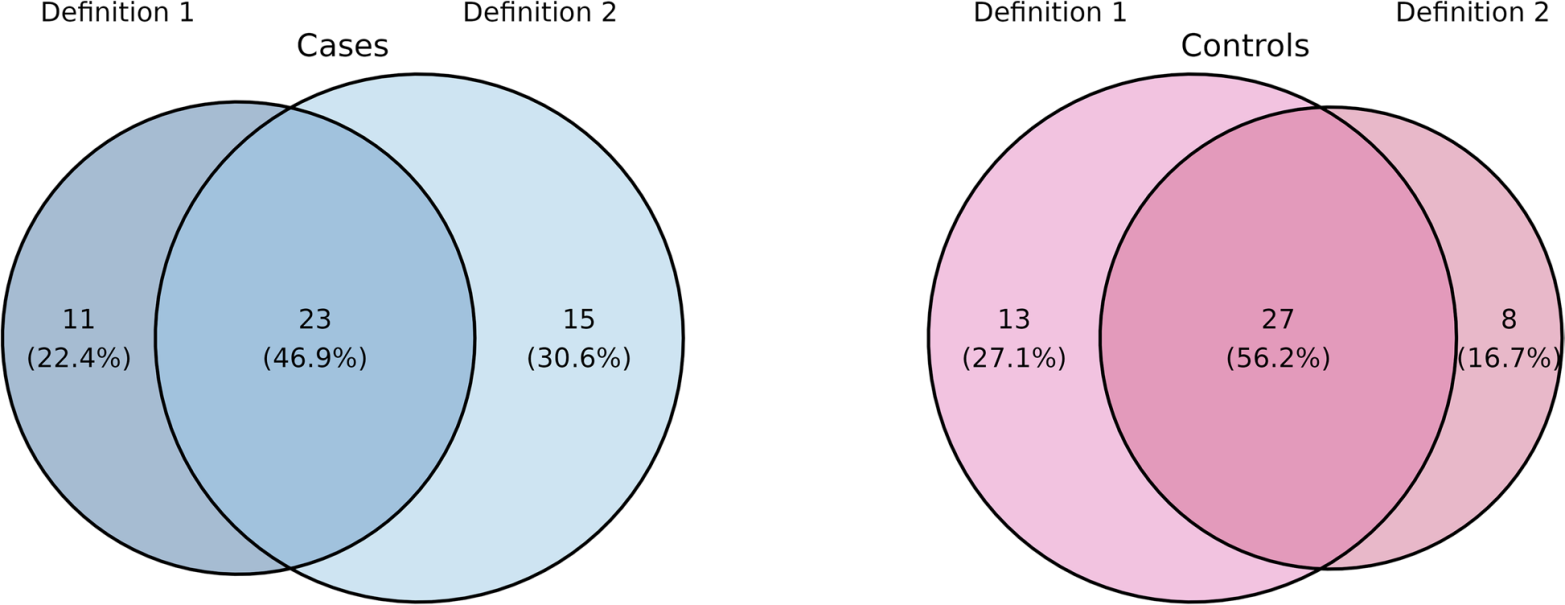
Number and percentage of genera in the core of cases (left, blue) and controls (right, pink), following definition 1 (prevalence and abundance) compared to definition 2 (hubs), depicting the intersection and distinct sets

Following definition 1, the core microbiota comprised 34 genera for cases and 40 genera for controls. To align the total number of core members in cases and controls for definition 2, we adjusted the quantile to 0.55 for defining hubs. This adjustment yielded 38 genera in the core for cases and 35 in the core for controls, with 23 overlapping for cases and 27 for controls with those core members defined by definition 1, as illustrated in Fig. 6. The specific core members can be found in Supplementary table S T2. We found for example unique core genera when using definition 2 like *Enterobacter*, *Enterococcus*, *Lactobacillus*, *Fusobacterium*, and *Morganella*. These genera are associated with dysbiosis in IBD. For instance, *Enterococcus* [60] and *Enterobacter* [61] have a higher resilience in inflamed or dysbiotic environments. This suggests that the centrality-based definition might capture genera more closely linked to pro-inflammatory states or dysbiosis, aligning with mechanisms relevant to IBD pathology.

**Fig. 7:**
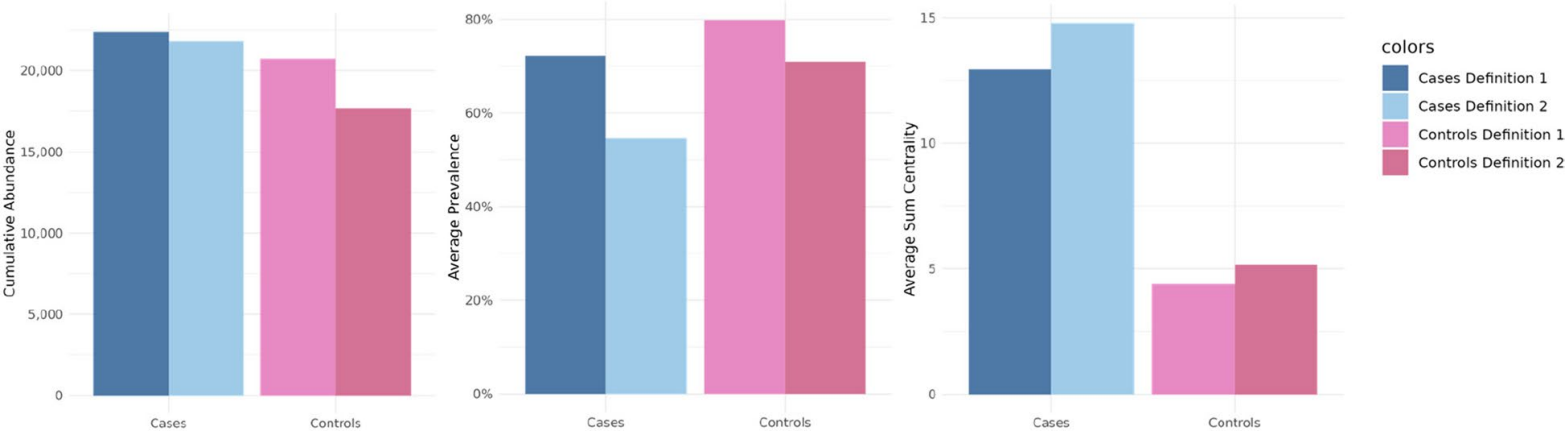
Comparing the cumulative abundance, average prevalence, and sum of all three centrality values, summed (averaged for centrality) over all core members identified by definition 1 (each left) and definition 2 (each right) for cases (blue) and controls (pink)

The cumulative abundance is higher using definition 1, which is based on abundance (cases: definition 1: 22,394, definition 2: 21,808, controls: definition1: 20,730, definition 2: 17,702). The average prevalence of core genera present per sample is between 55–80%, noticeable higher for definition 1 (cases: definition 1: 72%, definition 2: 55%, controls: definition 1: 80%, definition 2: 71%). The average over the sum of all three centrality measures was higher for core members found via definition 2, which is based on hubs and therefore on high centrality values, and it is in generally lower in controls (cases: definition 1: 13.2, definition 2: 14.9, controls: definition 1: 4.4, definition 2: 5.3). All those values are visualized in Fig. 7.

**Fig. 8:**
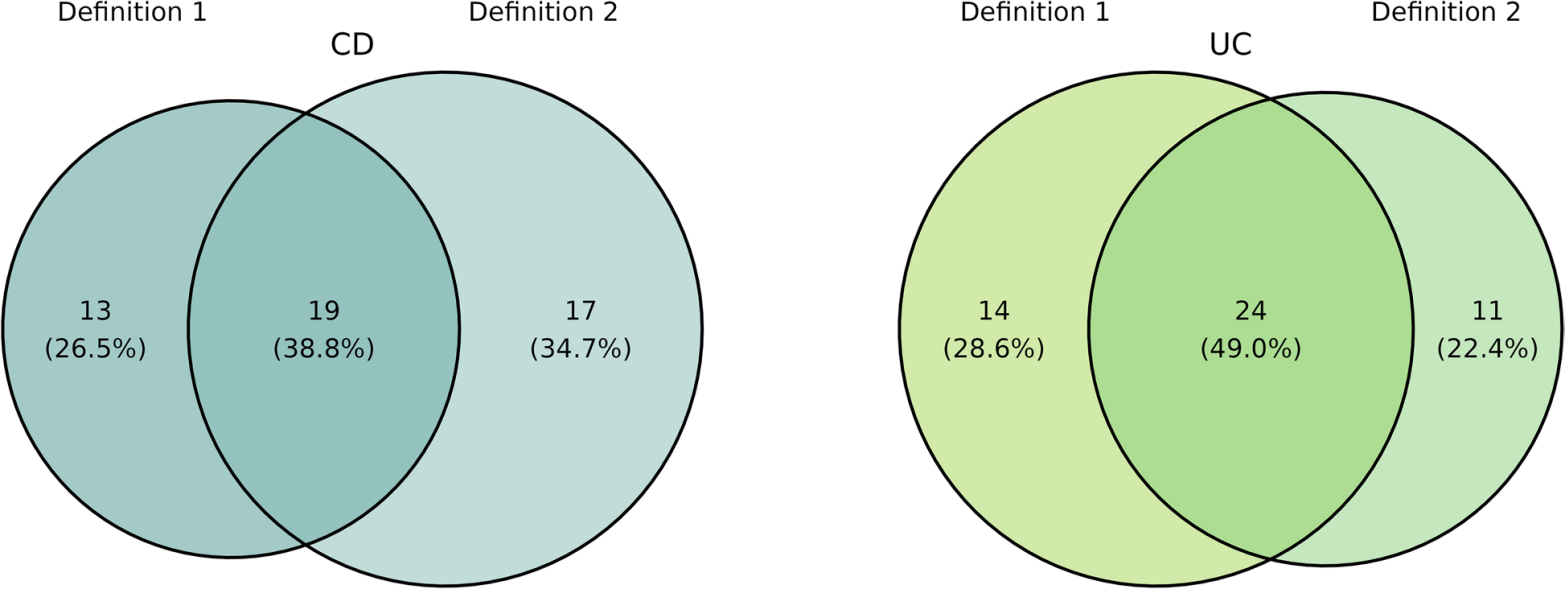
Number and percentage of genera in the core of CD (left, turquoise) and UC (right, light green) patients, following definition 1 (prevalence and abundance) compared to definition 2 (hubs), depicting the intersection and distinct sets

Doing the same comparisons for CD and UC patients, the core microbiota included 32 genera for CD and 38 genera for UC using definition 1. The quantile to define hubs was set to 0.575. Following definition 2, there were 36 genera in the core for CD and 35 genera in the core for UC patients, with 19 overlapping for CD and 24 overlapping for UC with those core members defined by definition 1, as illustrated in Fig. 8. A detailed list of core genera is given in Supplementary table S T3.

**Fig. 9:**
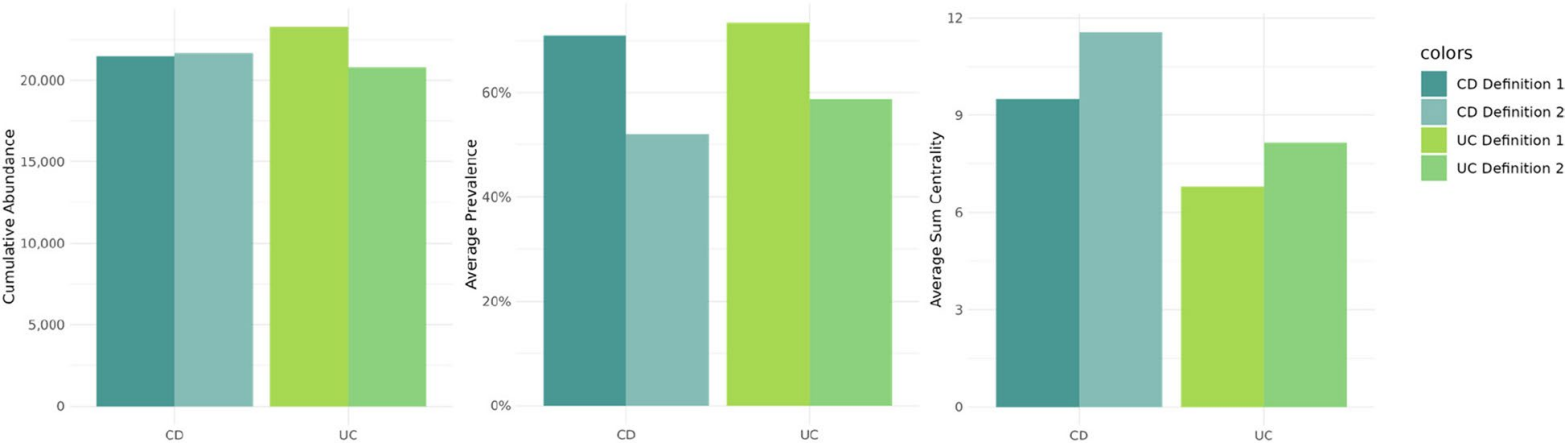
Comparing the cumulative abundance, average prevalence, and sum of all three centrality values, summed (averaged for centrality) over all core members identified by definition 1 (each left) and definition 2 (each right) for CD (turquoise) and UC (light green) patients

The cumulative abundance is very similar, independent of the used definition (CD: definition 1: 21,457, definition 2: 21,679, UC: definition 1: 23,252, definition 2: 20,803). The average prevalence of core taxa is noticeably higher when using definition 1 (CD: definition 1: 72%, definition 2: 5%, UC: definition 1: 80%, definition 2: 71%). Summing over of all three centrality measures for each genus and averaging over all core genera, using definition 2 yielded notably higher values for both CD and UC core genera (CD: definition 1: 9.5, definition 2: 11.6, UC: definition 1: 6.8, definition 2: 8.2). These values are visualized in Fig. 9.

### Role of individual nodes/Graphlet analysis/GCM’s

**Fig. 10:**
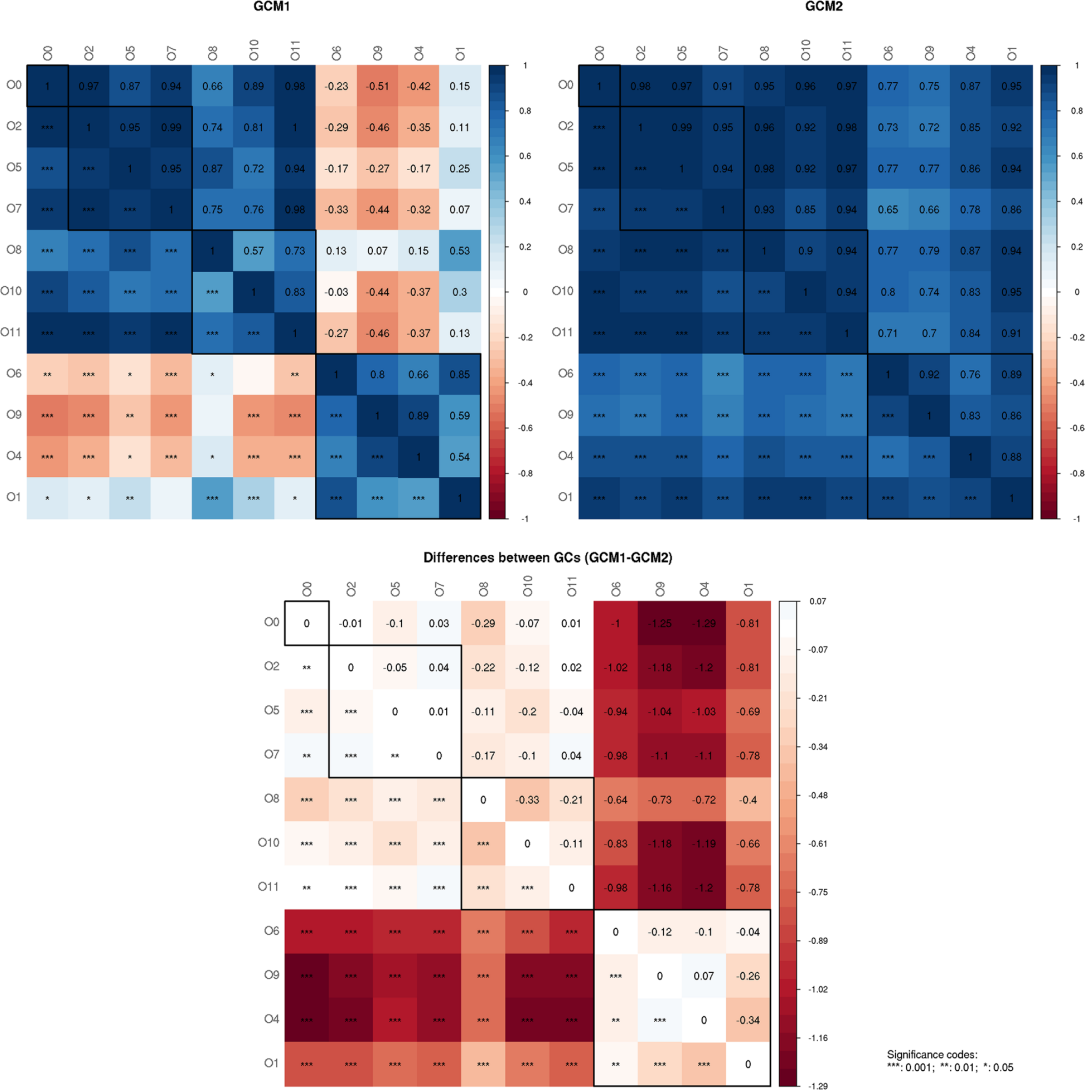
Presented are two graphlet correlation matrices computed for two different networks (based on cases (left GCM1) and controls (right GCM2)). The graphlet correlation matrix quantifies the pairwise similarity (Spearman correlation coefficient) of subgraph patterns (network's node orbits) in each network. In both cases, the color of each square represents the correlation coefficient between the corresponding pairs of graphlets. The diagonal of the matrices corresponds to the self-correlations of each graphlet. The matrix at the bottom shows the absolute difference between the graphlet correlation matrices for both networks. Positive values (blue) in the difference matrix indicate an increase in similarity, while negative values (red) indicate a decrease. Orbits are sorted according to their topological role

We calculated Graphlet correlation matrices (GCMs) from the constructed networks separately for cases, controls, CD, and UC. For comparisons between groups, we also computed a matrix containing the absolute difference between the GCMs for cases and controls (Fig. 10), as well as for CD and UC (Figure S2).

Our analysis revealed highly significant differences in the pairwise similarity of subgraph patterns for orbits 1, 4, 6, and 9. For those orbits, the Spearman correlation coefficients across node orbits differ significantly for the comparison of cases and controls, and differ slightly for the comparison between CD and UC. This is of particular interest, because all of these orbits have the same topological role: they represent end node orbits.

By mapping genera back to these orbits and focusing on the 25 most abundant ones in each of the four orbits, we identified sets of terminal nodes present in all orbits for inter-group comparisons listed in Table 5. Terminal nodes in an undirected graph represent nodes with just one single edge We also compared these terminal nodes to identified hub nodes. Notably, four genera appear in both lists, albeit in distinct disease states. *Bacteroides*, a hub for controls, appeared as a terminal node in UC patients. Similarly, *Clostridium XIVa*, identified as a hub for controls, is found as a terminal node for CD patients. *Faecalibacterium*, an important hub for IBD and both subtypes, appeared as a terminal node for controls. Additionally, *Subdoligranulum*, identified as a hub in CD patients, is observed as a terminal node in controls. Furthermore, we concentrated on terminal nodes exclusive to cases or controls and those exclusive to CD or UC. The averaged genus occurrence o (averaged over orbits 1,4,6, and 9) denotes how often a genus is part of substructures in accordance with the end node orbits. Terminal nodes exclusive to controls included *Clostridium XlVb* (o = 365), *Catabacter* (o = 330), *Streptococcus* (o = 322), *Faecalibacterium* (o = 304 *Subdoligranulum* (o = 283), *Mitsuokella* (o = 268), *Holdemania* (o = 285), and *Intestimonas* (o = 296). Conversely, genera exclusive to cases comprised *Dorea* (o = 3145), *Butyrivibrio* (o = 3322), and *Allisonella* (o = 3405). In CD, but not UC, genera included *Enterobacter* (o = 1673), *Hafnia* (o = 1656), *Fusicatenibacter* (o = 1264), *Clostridium XIVa* (o = 1697), *Butyricimonas* (o = 498), *Collinsella* (o = 1640), and *Bilophila* (o = 1540). Conversely, in UC, but not CD, genera encompassed *Bacteroides* (o = 917), *Catabacter* (o = 192), *Ruminococcus* (o = 855), *Parasutterella* (o = 921), and *Anaerovorax* (o = 914).

**Table 5:** Terminal nodes

Terminal nodes	controls	cases	CD	UC
*Allisonella*	81	**3405**	951	0
*Anaerovorax*	252	3386	1652	**914**
*Bacteroides*	240	2488	1439	**917**
*Bilophila*	186	2906	**1540**	828
*Butyricimonas*	261	3054	**498**	664
*Butyrivibrio*	10	**3322**	1575	452
*Catabacter*	**330**	3279	744	**192**
*Clostridium_XlVa*	229	2946	**1697**	838
*Clostridium_XlVb*	**365**	1353	337	83
*Collinsella*	236	3160	**1640**	765
*Dorea*	206	**3145**	1689	154
*Enterobacter*	56	1116	**1673**	728
*Faecalibacterium*	**304**	856	614	661
*Fusicatenibacter*	**293**	**3318**	**1264**	496
*Hafnia*	0	2939	**1656**	693
*Holdemania*	**285**	3335	1214	816
*Intestinimonas*	**296**	2344	1184	770
*Mitsuokella*	**268**	2970	407	808
*Parasutterella*	136	3374	1665	**921**
*Ruminococcus*	272	1622	931	**855**
*Streptococcus*	**322**	1510	756	796
*Subdoligranulum*	**283**	986	497	619

Terminal nodes for controls, cases, CD, and UC in alphabetical order, and averaged genus occurrences over orbits of interest (O1, O4, O6, and O9). Genera in bold indicate that those genera are terminal nodes in the respective group (column). Zero values indicate that this genus was an unconnected node in the group specific network. Additionally, the values from genera that are hubs in the previous analysis part are underlined

## Discussion

Examining global network properties of the gut microbiota, we found a more robust network structure in healthy controls, characterized by a higher number of components, and a greater presence of specialized, unconnected parts (components) compared to IBD patients. The higher edge number observed in controls suggests that the healthy population may harbor a more diverse range of microbial communities with specialized roles, supporting this hypothesis. Our findings are consistent with those reported by others [62], finding higher connectedness and lower centralization in healthy networks, implying a lower susceptibility to dysbiosis and an increased robustness in healthy individuals compared to IBD patients. Our analysis extends these observations by utilizing a larger cohort and by investigating a broader range of global network properties than has been explored in earlier studies. Moreover, our analysis revealed a higher clustering coefficient in the network of IBD cases, indicating an increase in co-dependence among microbial taxa within the whole network, which may negatively influence network stability. This finding is in line with previous results [63], of higher clustering coefficients in networks associated with various gastric conditions. Additionally, our study identified lower modularity in the networks of IBD cases, suggesting a loss of community structure and increased homogenization associated with disease pathology. This finding echoes the results of Baldassano et al. [64], who reported a lower modularity index in networks of IBD patients, indicating disruption to the communities of co-occurring organisms in the gut microbiota under disease conditions. Further research using perturbation theory, ideally with adjustment for covariates when available, is needed to verify that these global network properties result in a more robust structure for the healthy controls.

For CD and UC, global network properties were very similar, but significant differences emerged for local network properties.

Furthermore our analysis revealed significant diversity differences among IBD patients, with CD exhibiting lower diversity than UC, as observed in previous studies [65, 66]. Notably, no significant differences in alpha diversity were found between healthy controls and UC patients, consistent with prior research [67, 68]. This aligns with our findings comparing global network properties between UC and controls, and between CD and controls, where we observed no significant differences for UC but several for CD. These results are consistent with existing knowledge [69, 70] suggesting that CD is associated with a more strongly altered microbiota composition. However, differences in network properties between CD and controls have not been commonly reported, making our findings a valuable contribution to the understanding of the human gut microbiome in these conditions. When comparing CD and UC, it is important to consider differences arising from disease location, as particular ileal involvement may be a key driver. However, with only stool samples available, we can not empirically investigate the effect of disease location.

Examining local network properties, we identified hub nodes based on centrality measures, revealing distinct patterns between IBD and its subtypes compared to controls. While research exploring hubs for genes and pathways is available [29, 71], research on microbiota genera as hubs is still relatively scarce. This study addresses this gap, and for instance highlighted *Faecalibacterium*, an important butyrate producer [72], as a notable hub in IBD cases, despite its low abundance, indicating the importance of specific low-abundant genera, like other studies [73]. This observation resonates with further findings [74] in an investigation of Myalgic encephalomyelitis/chronic fatigue syndrome patients, where *Faecalibacterium* exhibited elevated centrality values in case networks across various metrics. In another study on the nasal microbiome of Chilean children with asthma [75], *Veillonella* and *Granulicatella* emerged as hubs in cases. This is comparable to our study, where *Veillonella* emerged as a hub in the network of IBD and both subtypes and *Granulicatella* emerged as a hub in the network of UC patients. This aligns with the biological role of *Veillonella*, an oral microbe that derives energy by fermenting short-chain organic acids. *Veillonella* utilizes nitrate, a metabolite enriched during inflammation, to grow, making it more abundant in the microbiome of IBD patients [76]. Another oral microbe, *Klebsiella,* emerged as a hub in cases, consistent with other findings [77] that demonstrate *Klebsiella’s* ability to induce Th1 cell responses, thereby promoting colitis. In contrast, hubs exclusive to controls shed light on potential protective mechanisms. Our analysis at the genus level identified *Bacteroides*, *Blautia*, *Clostridium XIVa*, and *Clostridium XVIII* as hubs uniquely in controls. This parallels findings from Izuno et al. [78] in their study on irritable bowel syndrome, where *Clostridium XIVa* exhibited hub characteristics in controls based on higher degree centrality. Additionally, another study on IBD at the species level [79] revealed species-specific hub dynamics, with *Bacteroides nordii* and *Bacteroides plebeius* emerging as hubs in controls and cases, respectively. Furthermore, Guo et al. [74] also identified *Blautia* as a hub in controls, consistent with our results.

When analyzing global and local network properties, it is important to raise awareness that the construction and analysis of networks involve many choices: starting at how to pre-process data, over which similarity measure to use, to choosing filters for nodes and cut-off values for edges of interest. We tried to stay within the most commonly used methods; however, we can not ensure that these are the most appropriate ones. For example, we employed SparCC due to its proven effectiveness in managing compositional bias and its computational efficiency for large-scale microbiome data [32]. However, recent advances, such as SPIEC-EASI, may provide improved network precision by focusing on direct associations, as noted by Kurtz et al. [58]. Both SparCC and SPIEC-EASI have shown strong performance in microbial network inference, yet each method has limitations depending on the data characteristics. Given the lack of a true gold-standard network, future studies might benefit from a combined or comparative approach to assess the robustness of microbial association networks more comprehensively. Another limitation in our network construction process and with the interpretation of results is the complexity of incorporating covariates, which prevented us from stratifying by factors such as age or BMI in this study. Moreover, in case–control comparisons, adjusting for covariates like inflammation or medication use is not straightforward, because many covariates of interest are strongly related to disease status. Future studies with standardized metadata should investigate alternative network approaches to integrate covariates more directly.

We utilized hub nodes to propose an alternative definition of a core microbiota in both cases and controls, as well as separately in patients with CD and UC. Core microbiota definitions vary widely, encompassing approaches such as community composition, functional profile, stability, and network-based methods [80]. We compared two definitions: one based on cut-off values for abundance and prevalence (definition 1), and another based on hub nodes as core members (definition 2). Notably, the latter requires only the selection of a quantile for hubs, unlike the former which necessitates the choice of at least two arbitrary thresholds [81].

A comparative analysis of core members under both definitions yielded comparable results. As expected, the average prevalence of core members was higher with definition 1, while the average sum of centrality values showed higher values when using definition 2. However, the cumulative abundance was almost independent of the definition used. Differences were also notable in terms of core members, particularly among cases (47% intersection) and CD patients (39% intersection). The overlap of core genera between both definitions is notifiable lower in cases and CD than in controls and UC. Dysbiotic states, especially in CD and cases, appear to shift the centrality values of certain genera. Some IBD-associated taxa may acquire higher centrality, even if their abundance or prevalence does not increase proportionally. Similar observations were made by Pisani et al. [82], where network members indicative of IBD exhibited larger centrality values. In conclusion, we advocate the use of hub nodes in defining the core microbiota. The advantage of using this centrality-based hubs definition is that it can incorporate taxa that may be low abundant but still biologically important. A potential limitation is that it may overlook taxa that are less connected in the network and thus exhibit lower centrality values. The use of hub nodes to define the core microbiota has already been used in other microbiome studies, such as fungal pollen microbiome [83] and plant microbiome [84]. However, in the human gut microbiome, applying this approach in direct comparison to traditional abundance- or prevalence-based methods appears to be relatively unexplored, and our study contributes to addressing this gap. Furthermore, the core microbiota could serve as a foundational element for manipulating the gut microbiome, potentially as a therapeutic target. So far, this application has been primarily explored in mice [85] and kelp [86], for instance, through the administration of prebiotics [87].

While the IBD-KC cohort provides a very large dataset, only 16S rRNA gene data was available for the present analysis. 16S data compared to e.g. whole-genome shotgun metagenomics is limited in its resolution [88], which might prevent some genera from appearing as hubs, such as *Akkermansia muciniphila* which has been shown to be relevant in IBD. Working at genus level provides a good balance between resolution and practicality when limited to 16S rRNA data. Furthermore, the analysis was based solely on microbiome data, limiting the scope of insights into microbial relationships. Incorporating other omics layers, such as proteomics, metagenomics, and transcriptomics, through multi-omics techniques could provide a more comprehensive understanding of the microbiome’s role in IBD. Similarly, replication in an independent dataset would be highly valuable to strengthen our findings. However, we evaluated what is, to our knowledge, the largest publicly available dataset (HMP2) and found that, in addition to being based on biopsy samples rather than stool samples, the remaining sample sizes after necessary filtering were too small to support a rigorous reanalysis using our network-based framework. Additionally, we only used genetically unrelated participants. Using the same family-based dataset with family controls and completely unrelated healthy controls, recent findings in metagenomic analysis [89] suggest that dividing unaffected controls into genetically related and unrelated groups could uncover additional differences. While we stratified samples based on clinical diagnosis, we acknowledge that dysbiosis is a dynamic process. Not all IBD patients may have experienced an active flare at the time of sampling. Therefore, especially the case group includes heterogeneity.

Our analysis not only emphasizes the significance of hub genera but also underscores the importance of terminal nodes, representing two extremes in local network properties. Terminal nodes, despite not necessarily being associated with low centrality, provide complementary insights to hubs and are equally important. Due to computational constraints limiting our graphlet analysis to $k=4$ nodes, hubs will not appear in these structures. Existing literature in microbiome and graphlet studies typically employs graphlet correlation distance for an overall comparison of microbial networks [90, 91]. However, exploration at the node level and mapping identified differences back to the involved taxa remains limited, rendering our approach new. We observed significant differences in orbits 1, 4, 6, and 9, which contain terminal nodes, between all group comparisons. Biological interpretations of terminal nodes in 16S microbiome data can be challenging, but is based on the idea that nodes in the same orbits have similar biological functions too [55, 56]. In the context of for example metabolic functions, terminal nodes often represent taxa involved in the final stages of metabolite processing. An example is the fermentation of dietary fiber to produce short-chain fatty acids [92], which are not further metabolized by other microbes but play a critical role in host health. In microbiome networks, terminal nodes may signify specialized taxa with unique functional roles. These taxa contribute to diversity and stability through their specialized activities, despite having limited connectivity within the microbial community.

We noted that some genera, identified as hubs in one disease state, acted as terminal nodes in another disease state. The dominant genera *Bacteroides* and the butyrate producer *Clostridium XIVa*, both having beneficial metabolic functions and higher abundance in healthy controls [93, 94], transitioned from being hubs in controls to terminal nodes in UC and CD, respectively. This transition aligns with the expectation that highly connected genera in controls exhibit reduced connectivity in cases. Similar results were found previously [95], finding different degree distributions between healthy controls and IBD cases, with higher degree values in the network of controls. *Faecalibacterium* and *Subdoligranulum*, both known to be short-chain fatty acid producers and identified as hubs in cases, were found to be terminal nodes for controls despite their typical higher abundance in controls [96]. This highlights again that high abundance does not always correspond to important local network properties. The observation that some genera transition from being terminal nodes in controls to hubs in IBD suggests a shift in microbial relationships in disease states. One possible explanation is that in the healthy state, these genera might play a more passive role in the network, supporting overall microbial homeostasis without the need for strong interconnections. However, in IBD, microbial dysbiosis could drive these genera to adopt a more central role, potentially as compensatory mechanisms in response to disease-related disturbances. For instance, *Faecalibacterium*, might have emerged as a hub in cases in response to the inflammatory milieu in IBD. Further analysis revealed terminal nodes unique to controls and those distinguishing CD from UC, with patterns more aligned with abundance. For example, *Streptococcus* and *Intestimonas*, known to be more abundant in cases [97, 98], were terminal nodes unique to controls. Additionally, based on the topological role of nodes, certain genera could be used to distinguish CD from UC. For instance, *Collinsella*, a terminal node only in CD, is consistent with its reported higher abundance in UC cases [69], and *Bacteroides*, a terminal node exclusively in UC, is consistent with findings of significantly increased abundance in CD patients [99].

## Conclusion

Our network-based study revealed significant differences in the global network properties of the gut microbiota between Inflammatory Bowel Disease (IBD) patients and healthy controls, indicating a potentially more robust network structure in healthy controls. Local properties varied among all groups, including subgroups of patients with Crohn's Disease (CD) and Ulcerative Colitis (UC), highlighting the important role of microbiota genera in IBD, particularly concerning hubs and terminal nodes. Results should be interpreted with caution given that many factors influence microbiome data, such as host-related covariates, and their generalizability would benefit from validation in external datasets. Additionally, we recommend considering the proposed core microbiota definition based on hub nodes.

## Supplementary Information

Supplementary Material 1.

## Data Availability

The 16S rRNA gene abundance data, as well as covariables from the IBD-KC cohort are available upon request from P2N biobank. Access token: P2N_4Z3RY; http://www.uksh.de/p2n/. P2N is a controlled-access human data repository subject to European data protection laws. Therefore, data access is subject to an application, ethics approval by the applicant’s ethics board and a data access agreement. The R code used for the main analysis is available at: https://github.com/IMIS-MedicalStatistics/Human-gut-microbiome-in-IBD-a-network-based-approach.git.

## Abbreviations

CD: Crohn's Disease
GCM: Graphlet correlation matrix
IBD: Inflammatory bowel disease
IBD-KC: Inflammatory bowel disease Kindred cohort
LCC: Largest connected component
UC: Ulcerative Colitis

## References

[1] Torres J, Mehandru S, Colombel J-F, Peyrin-Biroulet L. Crohn’s disease. The Lancet. 2017;389:1741–55. 10.1016/S0140-6736(16)31711-1 27914655

[2] Ungaro R, Mehandru S, Allen PB, Peyrin-Biroulet L, Colombel J-F. Ulcerative colitis. The Lancet. 2017;389:1756–70. 10.1016/S0140-6736(16)32126-2 PMC6487890 27914657

[3] Wehkamp J, Götz M, Herrlinger K, Steurer W, Stange EF. Inflammatory Bowel Disease Dtsch Arzteblatt Int. 2016;113:72–82. 10.3238/arztebl.2016.0072 PMC4782273 26900160

[4] Nijakowski K, Rutkowski R, Eder P, Simon M, Korybalska K, Witowski J, et al. Potential Salivary Markers for Differential Diagnosis of Crohn’s Disease and Ulcerative Colitis. Life. 2021;11:943. 34575091 10.3390/life11090943 PMC8469159

[5] Le Berre C, Ananthakrishnan AN, Danese S, Singh S, Peyrin-Biroulet L. Ulcerative Colitis and Crohn’s Disease Have Similar Burden and Goals for Treatment. Clin Gastroenterol Hepatol. 2020;18:14–23. 31301452 10.1016/j.cgh.2019.07.005

[6] Alatab S, Sepanlou SG, Ikuta K, Vahedi H, Bisignano C, Safiri S, et al. The global, regional, and national burden of inflammatory bowel disease in 195 countries and territories, 1990–2017: a systematic analysis for the Global Burden of Disease Study 2017. Lancet Gastroenterol Hepatol. 2020;5:17–30. 31648971 10.1016/S2468-1253(19)30333-4 PMC7026709

[7] Khan I, Ullah N, Zha L, Bai Y, Khan A, Zhao T, et al. Alteration of Gut Microbiota in Inflammatory Bowel Disease (IBD): Cause or Consequence? IBD Treatment Targeting the Gut Microbiome. Pathogens. 2019;8:126. 31412603 10.3390/pathogens8030126 PMC6789542

[8] Younis N, Zarif R, Mahfouz R. Inflammatory bowel disease: between genetics and microbiota. Mol Biol Rep. 2020;47:3053–63. 32086718 10.1007/s11033-020-05318-5

[9] Carreras-Torres R, Ibáñez-Sanz G, Obón-Santacana M, Duell EJ, Moreno V. Identifying environmental risk factors for inflammatory bowel diseases: a Mendelian randomization study. Sci Rep. 2020;10:19273. 33159156 10.1038/s41598-020-76361-2 PMC7648100

[10] Wark G, Samocha-Bonet D, Ghaly S, Danta M. The Role of Diet in the Pathogenesis and Management of Inflammatory Bowel Disease: A Review. Nutrients. 2020;13:135. 33396537 10.3390/nu13010135 PMC7823614

[11] Morais LH, Schreiber HL, Mazmanian SK. The gut microbiota–brain axis in behaviour and brain disorders. Nat Rev Microbiol. 2021;19:241–55. 33093662 10.1038/s41579-020-00460-0

[12] Rohmann N, Geese T, Nestel S, Schlicht K, Geisler C, Türk K, et al. Metabolic and lifestyle factors accelerate disease onset and alter gut microbiome in inflammatory non-communicable diseases. BMC Med. 2024;22:493. 39449123 10.1186/s12916-024-03709-0 PMC11515311

[13] Gevers D, Kugathasan S, Denson LA, Vázquez-Baeza Y, Van Treuren W, Ren B, et al. The Treatment-Naive Microbiome in New-Onset Crohn’s Disease. Cell Host Microbe. 2014;15:382–92. 24629344 10.1016/j.chom.2014.02.005 PMC4059512

[14] Zheng J, Sun Q, Zhang M, Liu C, Su Q, Zhang L, et al. Noninvasive, microbiome-based diagnosis of inflammatory bowel disease. Nat Med. 2024. 10.1038/s41591-024-03280-4. 39367251 10.1038/s41591-024-03280-4 PMC11645270

[15] Sorbara MT, Pamer EG. Microbiome-based therapeutics. Nat Rev Microbiol. 2022;20:365–80. 34992261 10.1038/s41579-021-00667-9

[16] Tomkins JE, Manzoni C. Advances in protein-protein interaction network analysis for Parkinson’s disease. Neurobiol Dis. 2021;155: 105395. 34022367 10.1016/j.nbd.2021.105395

[17] Abbas M, Matta J, Le T, Bensmail H, Obafemi-Ajayi T, Honavar V, et al. Biomarker discovery in inflammatory bowel diseases using network-based feature selection. PLoS ONE. 2019;14:e0225382. 31756219 10.1371/journal.pone.0225382 PMC6874333

[18] Neu AT, Allen EE, Roy K. Defining and quantifying the core microbiome: Challenges and prospects. Proc Natl Acad Sci. 2021;118:e2104429118. 34862327 10.1073/pnas.2104429118 PMC8713806

[19] Man WH, De Steenhuijsen Piters WAA, Bogaert D. The microbiota of the respiratory tract: gatekeeper to respiratory health. Nat Rev Microbiol. 2017;15:259–70. 28316330 10.1038/nrmicro.2017.14 PMC7097736

[20] Ruiz Castro PA, Yepiskoposyan H, Gubian S, Calvino-Martin F, Kogel U, Renggli K, et al. Systems biology approach highlights mechanistic differences between Crohn’s disease and ulcerative colitis. Sci Rep. 2021;11:11519. 34075172 10.1038/s41598-021-91124-3 PMC8169754

[21] Ashtiani M, Salehzadeh-Yazdi A, Razaghi-Moghadam Z, Hennig H, Wolkenhauer O, Mirzaie M, et al. A systematic survey of centrality measures for protein-protein interaction networks. BMC Syst Biol. 2018;12:80. 30064421 10.1186/s12918-018-0598-2 PMC6069823

[22] Ma B, Wang H, Dsouza M, Lou J, He Y, Dai Z, et al. Geographic patterns of co-occurrence network topological features for soil microbiota at continental scale in eastern China. ISME J. 2016;10:1891–901. 26771927 10.1038/ismej.2015.261 PMC5029158

[23] Berry D, Widder S. Deciphering microbial interactions and detecting keystone species with co-occurrence networks. Front Microbiol. 2014;5:219. 10.3389/fmicb.2014.00219 PMC4033041 24904535

[24] Kardos O, London A, Vinkó T. Stability of network centrality measures: a numerical study. Soc Netw Anal Min. 2020;10:80.

[25] Sharma A, Junge O, Szymczak S, Rühlemann MC, Enderle J, Schreiber S, et al. Network-based quantitative trait linkage analysis of microbiome composition in inflammatory bowel disease families. Front Genet. 2023;14:1048312. 36755569 10.3389/fgene.2023.1048312 PMC9901208

[26] Hočevar T, Demčar J. Computation of Graphlet Orbits for Nodes and Edges in Sparse Graphs. J Stat Softw. 2016;71:1–24.

[27] Pržulj N. Biological network comparison using graphlet degree distribution. Bioinformatics. 2007;23:e177–83. 17237089 10.1093/bioinformatics/btl301

[28] Mah C, Jayawardana T, Leong G, Koentgen S, Lemberg D, Connor SJ, et al. Assessing the Relationship between the Gut Microbiota and Inflammatory Bowel Disease Therapeutics: A Systematic Review. Pathogens. 2023;12:262. 36839534 10.3390/pathogens12020262 PMC9965214

[29] Cao Q, Sun X, Rajesh K, Chalasani N, Gelow K, Katz B, et al. Effects of Rare Microbiome Taxa Filtering on Statistical Analysis. Front Microbiol. 2021;11:607325. 33510727 10.3389/fmicb.2020.607325 PMC7835481

[30] Zhou R, Ng SK, Sung JJY, Goh WWB, Wong SH. Data pre-processing for analyzing microbiome data– A mini review. Comput Struct Biotechnol J. 2023;21:4804–15. 37841330 10.1016/j.csbj.2023.10.001 PMC10569954

[31] Smirnova E, Huzurbazar S, Jafari F. PERFect: PERmutation Filtering test for microbiome data. Biostat Oxf Engl. 2019;20:615–31. 10.1093/biostatistics/kxy020 PMC6797060 29917060

[32] Friedman J, Alm EJ. Inferring Correlation Networks from Genomic Survey Data. PLoS Comput Biol. 2012;8:e1002687. 23028285 10.1371/journal.pcbi.1002687 PMC3447976

[33] Peschel S, Müller CL, von Mutius E, Boulesteix A-L, Depner M. NetCoMi: network construction and comparison for microbiome data in R. Brief Bioinform. 2021;22:bbaa290. 10.1093/bib/bbaa290 PMC8293835 33264391

[34] Clauset A, Newman MEJ, Moore C. Finding community structure in very large networks. Phys Rev E. 2004;70:066111. 10.1103/PhysRevE.70.066111 15697438

[35] Tan Y-T, Ou-Yang L, Jiang X, Yan H, Zhang X-F. Identifying Gene Network Rewiring Based on Partial Correlation. IEEE/ACM Trans Comput Biol Bioinform. 2022;19:513–21. 32750866 10.1109/TCBB.2020.3002906

[36] Kiani NA, Gomez-Cabrero D, Bianconi G, editors. Networks of Networks in Biology: Concepts, Tools and Applications. 1st edition. Cambridge University Press; 2021.

[37] Barrat A, Barthélemy M, Pastor-Satorras R, Vespignani A. The architecture of complex weighted networks. Proc Natl Acad Sci. 2004;101:3747–52. 15007165 10.1073/pnas.0400087101 PMC374315

[38] Faust K, Lima-Mendez G, Lerat J-S, Sathirapongsasuti JF, Knight R, Huttenhower C, et al. Cross-biome comparison of microbial association networks. Front Microbiol. 2015;6:1200. 10.3389/fmicb.2015.01200 PMC4621437 26579106

[39] Wasserman S, Faust K. Social Network Analysis: Methods and Applications. 1st edition. Cambridge University Press; 1994.

[40] Jun W, Barahona M, Yue-Jin T, Hong-Zhong D. Natural Connectivity of Complex Networks. Chin Phys Lett. 2010;27:078902.

[41] De Lange N. Geoinformatik in Theorie und Praxis: Grundlagen von Geoinformationssystemen, Fernerkundung und digitaler Bildverarbeitung. Berlin, Heidelberg: Springer Berlin Heidelberg; 2020.

[42] Biological Network Analysis. Elsevier; 2020.

[43] Freeman LC. Centrality in social networks conceptual clarification. Soc Netw. 1978;1:215–39.

[44] Freeman LC. A Set of Measures of Centrality Based on Betweenness. Sociometry. 1977;40:35.

[45] Sabidussi G. The centrality index of a graph. Psychometrika. 1966;31:581–603. 5232444 10.1007/BF02289527

[46] Zhang J, Luo Y. Degree Centrality, Betweenness Centrality, and Closeness Centrality in Social Network. In: Proceedings of the 2017 2nd International Conference on Modelling, Simulation and Applied Mathematics (MSAM2017). Bankog, Thailand: Atlantis Press; 2017.

[47] Fisher CK, Mehta P. Identifying Keystone Species in the Human Gut Microbiome from Metagenomic Timeseries Using Sparse Linear Regression. PLoS ONE. 2014;9:e102451. 25054627 10.1371/journal.pone.0102451 PMC4108331

[48] Banerjee S, Schlaeppi K, Van Der Heijden MGA. Keystone taxa as drivers of microbiome structure and functioning. Nat Rev Microbiol. 2018;16:567–76. 29789680 10.1038/s41579-018-0024-1

[49] Kajihara KT, Hynson NA. Networks as tools for defining emergent properties of microbiomes and their stability. Microbiome. 2024;12:184. 39342398 10.1186/s40168-024-01868-z PMC11439251

[50] Real R, Vargas JM. The Probabilistic Basis of Jaccard’s Index of Similarity. Syst Biol. 1996;45:380–5.

[51] McMurdie PJ, Holmes S. phyloseq: An R Package for Reproducible Interactive Analysis and Graphics of Microbiome Census Data. PLoS ONE. 2013;8: e61217. 23630581 10.1371/journal.pone.0061217 PMC3632530

[52] Leo Lahti SS. microbiome. 2017.

[53] Yaveroğlu ÖN, Malod-Dognin N, Davis D, Levnajic Z, Janjic V, Karapandza R, et al. Revealing the Hidden Language of Complex Networks. Sci Rep. 2014;4:4547. 24686408 10.1038/srep04547 PMC3971399

[54] Roux J, Bez N, Rochet P, Joo R, Mahévas S. Graphlet correlation distance to compare small graphs. PLoS ONE. 2023;18:e0281646. 36791120 10.1371/journal.pone.0281646 PMC9931116

[55] Zelezniak A, Andrejev S, Ponomarova O, Mende DR, Bork P, Patil KR. Metabolic dependencies drive species co-occurrence in diverse microbial communities. Proc Natl Acad Sci. 2015;112:6449–54. 25941371 10.1073/pnas.1421834112 PMC4443341

[56] Levy R, Borenstein E. Metabolic modeling of species interaction in the human microbiome elucidates community-level assembly rules. Proc Natl Acad Sci. 2013;110:12804–9. 23858463 10.1073/pnas.1300926110 PMC3732988

[57] Li Y, Wu F-X, Ngom A. A review on machine learning principles for multi-view biological data integration. Brief Bioinform. 2016;19:bbw113. 10.1093/bib/bbw113 28011753

[58] Kurtz ZD, Müller CL, Miraldi ER, Littman DR, Blaser MJ, Bonneau RA. Sparse and Compositionally Robust Inference of Microbial Ecological Networks. PLOS Comput Biol. 2015;11:e1004226. 25950956 10.1371/journal.pcbi.1004226 PMC4423992

[59] Valente TW, Coronges K, Lakon C, Costenbader E. How Correlated Are Network Centrality Measures? Connect Tor Ont. 2008;28:16–26. PMC2875682 20505784

[60] Růžičková M, Vítězová M, Kushkevych I. The characterization of *Enterococcus* genus: resistance mechanisms and inflammatory bowel disease. Open Med. 2020;15:211–24. 10.1515/med-2020-0032 PMC7147287 32292819

[61] Baldelli V, Scaldaferri F, Putignani L, Del Chierico F. The Role of Enterobacteriaceae in Gut Microbiota Dysbiosis in Inflammatory Bowel Diseases. Microorganisms. 2021;9:697. 33801755 10.3390/microorganisms9040697 PMC8066304

[62] Rausch P, Ellul S, Pisani A, Bang C, Tabone T, Marantidis Cordina C, et al. Microbial Dynamics in Newly Diagnosed and Treatment Naïve IBD Patients in the Mediterranean. Inflamm Bowel Dis. 2023;29:1118–32. 36735955 10.1093/ibd/izad004 PMC10320370

[63] Appiah EM, Yakubu B, Salifu SP. Comprehensive microbial network analysis of gastric microbiome reveal key species affecting gastric carcinogenesis. The Microbe. 2023;1:100009.

[64] Baldassano SN, Bassett DS. Topological distortion and reorganized modular structure of gut microbial co-occurrence networks in inflammatory bowel disease. Sci Rep. 2016;6:26087. 27188829 10.1038/srep26087 PMC4870640

[65] Halfvarson J, Brislawn CJ, Lamendella R, Vázquez-Baeza Y, Walters WA, Bramer LM, et al. Dynamics of the human gut microbiome in inflammatory bowel disease. Nat Microbiol. 2017;2:17004. 28191884 10.1038/nmicrobiol.2017.4 PMC5319707

[66] Sankarasubramanian J, Ahmad R, Avuthu N, Singh AB, Guda C. Gut Microbiota and Metabolic Specificity in Ulcerative Colitis and Crohn’s Disease. Front Med. 2020;7:606298. 10.3389/fmed.2020.606298 PMC7729129 33330572

[67] Herrera-deGuise C, Varela E, Sarrabayrouse G, Pozuelo Del Río M, Alonso VR, Sainz NB, et al. Gut Microbiota Composition in Long-Remission Ulcerative Colitis is Close to a Healthy Gut Microbiota. Inflamm Bowel Dis. 2023;29:1362–9. 37655859 10.1093/ibd/izad058

[68] Zakerska-Banaszak O, Tomczak H, Gabryel M, Baturo A, Wolko L, Michalak M, et al. Dysbiosis of gut microbiota in Polish patients with ulcerative colitis: a pilot study. Sci Rep. 2021;11:2166. 33495479 10.1038/s41598-021-81628-3 PMC7835370

[69] Pascal V, Pozuelo M, Borruel N, Casellas F, Campos D, Santiago A, et al. A microbial signature for Crohn’s disease. Gut. 2017;66:813–22. 28179361 10.1136/gutjnl-2016-313235 PMC5531220

[70] Dalal SR, Chang EB. The microbial basis of inflammatory bowel diseases. J Clin Invest. 2014;124:4190–6. 25083986 10.1172/JCI72330 PMC4191005

[71] Huang Y, Zhang X, PengWang, Li Y, Yao J. Identification of hub genes and pathways in colitis-associated colon cancer by integrated bioinformatic analysis. BMC Genomic Data. 2022;23:48. 10.1186/s12863-022-01065-7 PMC9219145 35733095

[72] Lloyd-Price J, Arze C, Ananthakrishnan AN, Schirmer M, Avila-Pacheco J, Poon TW, et al. Multi-omics of the gut microbial ecosystem in inflammatory bowel diseases. Nature. 2019;569:655–62. 31142855 10.1038/s41586-019-1237-9 PMC6650278

[73] Guo B, Zhang L, Sun H, Gao M, Yu N, Zhang Q, et al. Microbial co-occurrence network topological properties link with reactor parameters and reveal importance of low-abundance genera. Npj Biofilms Microbiomes. 2022;8:3. 35039527 10.1038/s41522-021-00263-y PMC8764041

[74] Guo C, Che X, Briese T, Ranjan A, Allicock O, Yates RA, et al. Deficient butyrate-producing capacity in the gut microbiome is associated with bacterial network disturbances and fatigue symptoms in ME/CFS. Cell Host Microbe. 2023;31:288-304.e8. 36758522 10.1016/j.chom.2023.01.004 PMC10183837

[75] Ramos-Tapia I, Reynaldos-Grandón KL, Pérez-Losada M, Castro-Nallar E. Characterization of the upper respiratory tract microbiota in Chilean asthmatic children reveals compositional, functional, and structural differences. Front Allergy. 2023;4:1223306. 37577334 10.3389/falgy.2023.1223306 PMC10419220

[76] Rojas-Tapias DF, Brown EM, Temple ER, Onyekaba MA, Mohamed AMT, Duncan K, et al. Inflammation-associated nitrate facilitates ectopic colonization of oral bacterium Veillonella parvula in the intestine. Nat Microbiol. 2022;7:1673–85. 36138166 10.1038/s41564-022-01224-7 PMC9728153

[77] Atarashi K, Suda W, Luo C, Kawaguchi T, Motoo I, Narushima S, et al. Ectopic colonization of oral bacteria in the intestine drives T _H_ 1 cell induction and inflammation. Science. 2017;358:359–65. 29051379 10.1126/science.aan4526 PMC5682622

[78] Izuno S, Yoshihara K, Sudo N. Role of Gut Microbiota in the Pathophysiology of Stress-Related Disorders: Evidence from Neuroimaging Studies. Ann Nutr Metab. 2021;77(Suppl. 2):4–10. 34280919 10.1159/000517420

[79] Ma Y, Zhang Y, Xiang J, Xiang S, Zhao Y, Xiao M, et al. Metagenome Analysis of Intestinal Bacteria in Healthy People, Patients With Inflammatory Bowel Disease and Colorectal Cancer. Front Cell Infect Microbiol. 2021;11:599734. 33738265 10.3389/fcimb.2021.599734 PMC7962608

[80] Sharon I, Quijada NM, Pasolli E, Fabbrini M, Vitali F, Agamennone V, et al. The Core Human Microbiome: Does It Exist and How Can We Find It? A Critical Review of the Concept. Nutrients. 2022;14:2872. 35889831 10.3390/nu14142872 PMC9323970

[81] Risely A, Gillingham MAF, Béchet A, Brändel S, Heni AC, Heurich M, et al. Phylogeny- and Abundance-Based Metrics Allow for the Consistent Comparison of Core Gut Microbiome Diversity Indices Across Host Species. Front Microbiol. 2021;12:659918. 34046023 10.3389/fmicb.2021.659918 PMC8144293

[82] Pisani A, Rausch P, Bang C, Ellul S, Tabone T, Marantidis Cordina C, et al. Dysbiosis in the Gut Microbiota in Patients with Inflammatory Bowel Disease during Remission. Microbiol Spectr. 2022;10:e0061622. 35532243 10.1128/spectrum.00616-22 PMC9241752

[83] Manirajan BA, Maisinger C, Ratering S, Rusch V, Schwiertz A, Cardinale M, et al. Diversity, specificity, co-occurrence and hub taxa of the bacterial–fungal pollen microbiome. FEMS Microbiol Ecol. 2018;94:fiy112. 10.1093/femsec/fiy112 29878113

[84] Agler MT, Ruhe J, Kroll S, Morhenn C, Kim S-T, Weigel D, et al. Microbial Hub Taxa Link Host and Abiotic Factors to Plant Microbiome Variation. PLOS Biol. 2016;14:e1002352. 26788878 10.1371/journal.pbio.1002352 PMC4720289

[85] Malfatti MA, Kuhn EA, Murugesh DK, Mendez ME, Hum N, Thissen JB, et al. Manipulation of the Gut Microbiome Alters Acetaminophen Biodisposition in Mice. Sci Rep. 2020;10:4571. 32165665 10.1038/s41598-020-60982-8 PMC7067795

[86] Park S, Zhang T, Kang S. Fecal Microbiota Composition, Their Interactions, and Metagenome Function in US Adults with Type 2 Diabetes According to Enterotypes. Int J Mol Sci. 2023;24:9533. 37298483 10.3390/ijms24119533 PMC10253423

[87] Fan S, Zhang Z, Zhao Y, Daglia M, Zhang J, Zhu Y, et al. Recent advances in targeted manipulation of the gut microbiome by prebiotics: from taxonomic composition to metabolic function. Curr Opin Food Sci. 2023;49:100959.

[88] Durazzi F, Sala C, Castellani G, Manfreda G, Remondini D, De Cesare A. Comparison between 16S rRNA and shotgun sequencing data for the taxonomic characterization of the gut microbiota. Sci Rep. 2021;11:3030. 33542369 10.1038/s41598-021-82726-y PMC7862389

[89] Rühlemann M, Waschina S, Wacker EM, Rausch P, Schaan A, Zafroon Z, et al. Disease signatures in the gut metagenome of a prospective family cohort of inflammatory bowel disease. preprint. Gastroenterology; 2023.

[90] Leung MHY, Tong X, Wilkins D, Cheung HHL, Lee PKH. Individual and household attributes influence the dynamics of the personal skin microbiota and its association network. Microbiome. 2018;6:26. 29394957 10.1186/s40168-018-0412-9 PMC5797343

[91] Mahana D, Trent CM, Kurtz ZD, Bokulich NA, Battaglia T, Chung J, et al. Antibiotic perturbation of the murine gut microbiome enhances the adiposity, insulin resistance, and liver disease associated with high-fat diet. Genome Med. 2016;8:48. 27124954 10.1186/s13073-016-0297-9 PMC4847194

[92] Blaak EE, Canfora EE, Theis S, Frost G, Groen AK, Mithieux G, et al. Short chain fatty acids in human gut and metabolic health. Benef Microbes. 2020;11:411–55. 32865024 10.3920/BM2020.0057

[93] Louis P, Flint HJ. Diversity, metabolism and microbial ecology of butyrate-producing bacteria from the human large intestine. FEMS Microbiol Lett. 2009;294:1–8. 19222573 10.1111/j.1574-6968.2009.01514.x

[94] Zhou Y, Zhi F. Lower Level of Bacteroides in the Gut Microbiota Is Associated with Inflammatory Bowel Disease: A Meta-Analysis. BioMed Res Int. 2016;2016:5828959. 27999802 10.1155/2016/5828959 PMC5143693

[95] Naqvi A, Rangwala H, Keshavarzian A, Gillevet P. Network-Based Modeling of the Human Gut Microbiome. Chem Biodivers. 2010;7:1040–50. 20491063 10.1002/cbdv.200900324 PMC3681515

[96] Chang T-E, Luo J-C, Yang U-C, Huang Y-H, Hou M-C, Lee F-Y. Fecal microbiota profile in patients with inflammatory bowel disease in Taiwan. J Chin Med Assoc. 2021;84:580–7. 33871395 10.1097/JCMA.0000000000000532 PMC12966102

[97] Heidarian F, Noormohammadi Z, Asadzadeh Aghdaei H, Alebouyeh M. Relative Abundance of Streptococcus spp. and its Association with Disease Activity in Inflammatory Bowel Disease Patients Compared with Controls. Arch Clin Infect Dis. 2017;In Press In Press.

[98] Kamp K, Plantinga AM, Cain KC, Burr R, Wu Q, So SY, et al. S624 The Gut Microbiome and Symptoms in Irritable Bowel Syndrome. Am J Gastroenterol. 2023;118:S458–S458.

[99] Andoh A, Imaeda H, Aomatsu T, Inatomi O, Bamba S, Sasaki M, et al. Comparison of the fecal microbiota profiles between ulcerative colitis and Crohn’s disease using terminal restriction fragment length polymorphism analysis. J Gastroenterol. 2011;46:479–86. 21253779 10.1007/s00535-010-0368-4

