# Supplementary material for: The human gut microbiota in IBD, characterizing hubs, the core microbiota and terminal nodes: a network-based approach
Source: BMC Microbiol. 2025 Jun 26;25:371. doi: 10.1186/s12866-025-04106-0 (PMC12199529; doi:10.1186/s12866-025-04106-0)
Supplement: Supplementary file 1 — Supplementary Material 1. [file 12866_2025_4106_MOESM1_ESM.docx]

Supplement/ Additional files

**Methods:** Gut microbiota

Fecal samples from study participants were collected at home using standard stool collection tubes and subsequently mailed to the study center. Samples were stored at −80°C until further processed by the Institute of Clinical Molecular Biology at Kiel University, Germany. DNA extraction from approximately 200 mg of fecal material was conducted using QIAamp DNA stool mini-kits, with automation on the QIAcube (QIAGEN). The subsequent steps involved 16S rRNA gene library preparation and sequencing. Specifically, the V1-V2 region of the 16S rRNA gene was sequenced on the MiSeq platform, employing v3 chemistry for 2 × 300 bp paired-end reads (Illumina Inc., San Diego, CA, United States). Data processing utilized the DADA2 version 1.10 workflow [1], resulting in Amplicon Sequence Variant (ASV) abundance tables. Different sequencing runs were managed separately for a V1-V2-adjusted workflow [2] and were consolidated into a single abundance table per dataset only after final chimera filtering. ASVs underwent taxonomic annotation using the Bayesian classifier provided by DADA2 and the Ribosomal Database Project (RDP) version 16 release. All subsequent microbiota analyses were conducted at the genus level.

## Data pre-processing

Subjects with missing values (NA) across all variables were excluded. Individuals without known sex were also excluded, resulting in the exclusion of 23 subjects. Additionally subjects with a BMI exceeding 35 kg/m2 were excluded, leading to the removal of 35 subjects. Incomplete data for clinical variables were addressed through imputation: Specifically, age for one individual, height for six individuals, and weight for eleven individuals were imputed using the median values specific to their respective groups (cases or controls). For smoking, missing values were replaced with 0 by default, indicating no current smoking habits (3 individuals). As we used the family-based dataset as a case-control dataset, we only considered the maximal set of unrelated individuals, applying the R package kinship2 and the function pedigree and pedigree.unrelated(). All these steps reduced the cohort size from 1715 to 887 individuals.

Statistical analysis

Statistical analysis and data visualization was performed with R (Ubuntu Jammy: R version 4.3.0), R Studio (Ubuntu Jammy: “Desert Sunflower” 2023.09.1), as well as using Jupyter notebook (version 6.4.8 of notebook server and Python (version 3.10.12)).

**S T1: Global network properties**

| **Global Network properties** | **UC** | **Controls** | ${p_{UC/controls}}$ | **CD** | **Controls** | ${p_{CD/controls}}$ |
| --- | --- | --- | --- | --- | --- | --- |
| Number of components | 10 | 12 | 1.0 | 2 | 10 | 0.09 |
| Clustering coefficient | 0.47 | 0.46 | 0.9 | 0.55 | 0.49 | 0.27 |
| Modularity | 0.24 | 0.34 | 0.45 | 0.17 | 0.33 | 0.09 |
| Positive edge percentage | 58.89 | 68.34 | 0.27 | 55.53 | 67.37 | 0.09 |
| Edge density | 0.13 | 0.08 | 0.9 | 0.22 | 0.09 | 0.09 |
| Natural connectivity | 0.036 | 0.023 | 0.81 | 0.066 | 0.024 | 0.09 |
| Edge number | 1.3 | 0.96 | n.a.^c^ | 0.44 | 0.9 | n.a.^c^ |
| **Largest connected component (LCC)** | | | | | | |
| Relative LCC size | 0.92 | 0.86 | 1.0 | 0.99 | 0.88 | 0.09 |
| Average dissimilarity ^a^ | 0.95 | 0.96 | 0.81 | 0.93 | 0.96 | 0.09 |
| Average path length ^b^ | 1.66 | 1.71 | 1.0 | 1.41 | 1.70 | 0.09 |
| Clustering coefficient | 0.47 | 0.46 | 0.9 | 0.55 | 0.49 | 0.27 |
| Modularity | 0.24 | 0.33 | 0.45 | 0.17 | 0.33 | 0.09 |

Comparison of global network properties between controls and cases, as well as between CD and UC patients. P-value obtained through a permutation test (10 permutations). ^a^ Dissimilarity = 1 - edge weight, ^b^ Path length = Units with average dissimilarity, ^c^ p-value not available, due to specific definition of edge number.

# S T2: Core members for cases and controls

| **controls definition 1** | **controls definition 2** | **cases definition 1** | **cases definition 2** |
| --- | --- | --- | --- |
| Akkermansia | Alistipes | Alistipes | Alistipes |
| Alistipes | Alloprevotella | Anaerostipes | Anaerotruncus |
| Anaerostipes | Anaerostipes | Anaerotruncus | Bacteroides |
| Anaerotruncus | Anaerotruncus | Bacteroides | Barnesiella |
| Anaerovorax | Anaerovorax | Barnesiella | Blautia |
| Bacteroides | Bacteroides | Bilophila | Butyricimonas |
| Barnesiella | Barnesiella | Blautia | Citrobacter |
| Bilophila | Blautia | Butyricicoccus | Clostridium_IV |
| Blautia | Butyricicoccus | Clostridium_IV | Clostridium_sensu_stricto |
| Butyricicoccus | Butyricimonas | Clostridium_sensu_stricto | Clostridium_XlVa |
| Butyricimonas | Catabacter | Clostridium_XlVa | Clostridium_XlVb |
| Catabacter | Catenibacterium | Clostridium_XlVb | Collinsella |
| Clostridium_IV | Clostridium_IV | Clostridium_XVIII | Coprobacter |
| Clostridium_sensu_stricto | Clostridium_XlVa | Collinsella | Enterobacter |
| Clostridium_XlVa | Clostridium_XlVb | Coprococcus | Enterococcus |
| Clostridium_XlVb | Clostridium_XVIII | Dialister | Escherichia.Shigella |
| Clostridium_XVIII | Coprobacter | Dorea | Faecalibacterium |
| Collinsella | Dorea | Eggerthella | Flavonifractor |
| Coprobacter | Faecalibacterium | Escherichia.Shigella | Fusobacterium |
| Coprococcus | Flavonifractor | Faecalibacterium | Intestinimonas |
| Dialister | Fusicatenibacter | Flavonifractor | Klebsiella |
| Dorea | Holdemanella | Fusicatenibacter | Lactobacillus |
| Eggerthella | Holdemania | Intestinibacter | Lactococcus |
| Escherichia.Shigella | Klebsiella | Odoribacter | Megasphaera |
| Faecalibacterium | Mitsuokella | Oscillibacter | Morganella |
| Flavonifractor | Odoribacter | Parabacteroides | Odoribacter |
| Fusicatenibacter | Oscillibacter | Parasutterella | Oscillibacter |
| Intestinimonas | Oxalobacter | Pseudoflavonifractor | Parabacteroides |
| Odoribacter | Prevotella | Roseburia | Proteus |
| Oscillibacter | Pseudoflavonifractor | Ruminococcus | Pseudoflavonifractor |
| Parabacteroides | Roseburia | Ruminococcus2 | Raoultella |
| Parasutterella | Ruminococcus | Streptococcus | Roseburia |
| Pseudoflavonifractor | Ruminococcus2 | Subdoligranulum | Ruminococcus |
| Romboutsia | Streptococcus | Veillonella | Ruminococcus2 |
| Roseburia | Subdoligranulum |  | Solobacterium |
| Ruminococcus |  |  | Streptococcus |
| Ruminococcus2 |  |  | Subdoligranulum |
| Streptococcus |  |  | Veillonella |
| Subdoligranulum |  |  |  |
| Sutterella |  |  |  |

List of core members at genus level for cases and controls following definition 1 (based on prevalence (50/100) and abundance (0.1/100)) and definition 2 (hub nodes, quantile = 0.55).

#

# S T3: Core members for CD and UC patients

| **CD definition 1** | **CD definition 2** | **UC definition 1** | **UC definition 2** |
| --- | --- | --- | --- |
| Alistipes | Actinomyces | Alistipes | Actinomyces |
| Anaerostipes | Alistipes | Anaerostipes | Alistipes |
| Anaerotruncus | Anaerotruncus | Anaerotruncus | Anaerotruncus |
| Bacteroides | Bacteroides | Anaerovorax | Anaerovorax |
| Bilophila | Barnesiella | Bacteroides | Bacteroides |
| Blautia | Blautia | Barnesiella | Barnesiella |
| Butyricicoccus | Butyricimonas | Bilophila | Blautia |
| Clostridium_IV | Citrobacter | Blautia | Catabacter |
| Clostridium_XlVa | Clostridium_IV | Butyricicoccus | Citrobacter |
| Clostridium_XlVb | Clostridium_sensu_stricto | Clostridium_IV | Clostridium_IV |
| Clostridium_XVIII | Clostridium_XlVa | Clostridium_sensu_stricto | Clostridium_sensu_stricto |
| Collinsella | Clostridium_XlVb | Clostridium_XlVa | Clostridium_XlVa |
| Coprococcus | Corynebacterium | Clostridium_XlVb | Clostridium_XlVb |
| Dialister | Enterococcus | Clostridium_XVIII | Enterobacter |
| Dorea | Escherichia.Shigella | Collinsella | Enterococcus |
| Eggerthella | Faecalibacterium | Coprococcus | Escherichia.Shigella |
| Escherichia.Shigella | Flavonifractor | Dialister | Faecalibacterium |
| Faecalibacterium | Fusobacterium | Dorea | Flavonifractor |
| Flavonifractor | Granulicatella | Eggerthella | Fusobacterium |
| Fusicatenibacter | Intestinimonas | Escherichia.Shigella | Granulicatella |
| Intestinibacter | Klebsiella | Faecalibacterium | Intestinimonas |
| Odoribacter | Lactobacillus | Flavonifractor | Klebsiella |
| Oscillibacter | Megasphaera | Fusicatenibacter | Odoribacter |
| Parabacteroides | Morganella | Haemophilus | Oscillibacter |
| Parasutterella | Odoribacter | Intestinibacter | Parabacteroides |
| Pseudoflavonifractor | Oscillibacter | Intestinimonas | Proteus |
| Roseburia | Parabacteroides | Odoribacter | Pseudoflavonifractor |
| Ruminococcus | Propionibacterium | Oscillibacter | Raoultella |
| Ruminococcus2 | Proteus | Parabacteroides | Romboutsia |
| Streptococcus | Pseudoflavonifractor | Parasutterella | Ruminococcus |
| Subdoligranulum | Raoultella | Pseudoflavonifractor | Ruminococcus2 |
| Veillonella | Ruminococcus | Romboutsia | Streptococcus |
|  | Ruminococcus2 | Roseburia | Subdoligranulum |
|  | Streptococcus | Ruminococcus | Terrisporobacter |
|  | Subdoligranulum | Ruminococcus2 | Veillonella |
|  | Veillonella | Streptococcus |  |
|  |  | Subdoligranulum |  |

List of core members at genus level for CD and UC patients following definition 1 (based on prevalence (50/100) and abundance (0.1/100)) and definition 2 (hub nodes, quantile = 0.575).

**Figure S1**

**
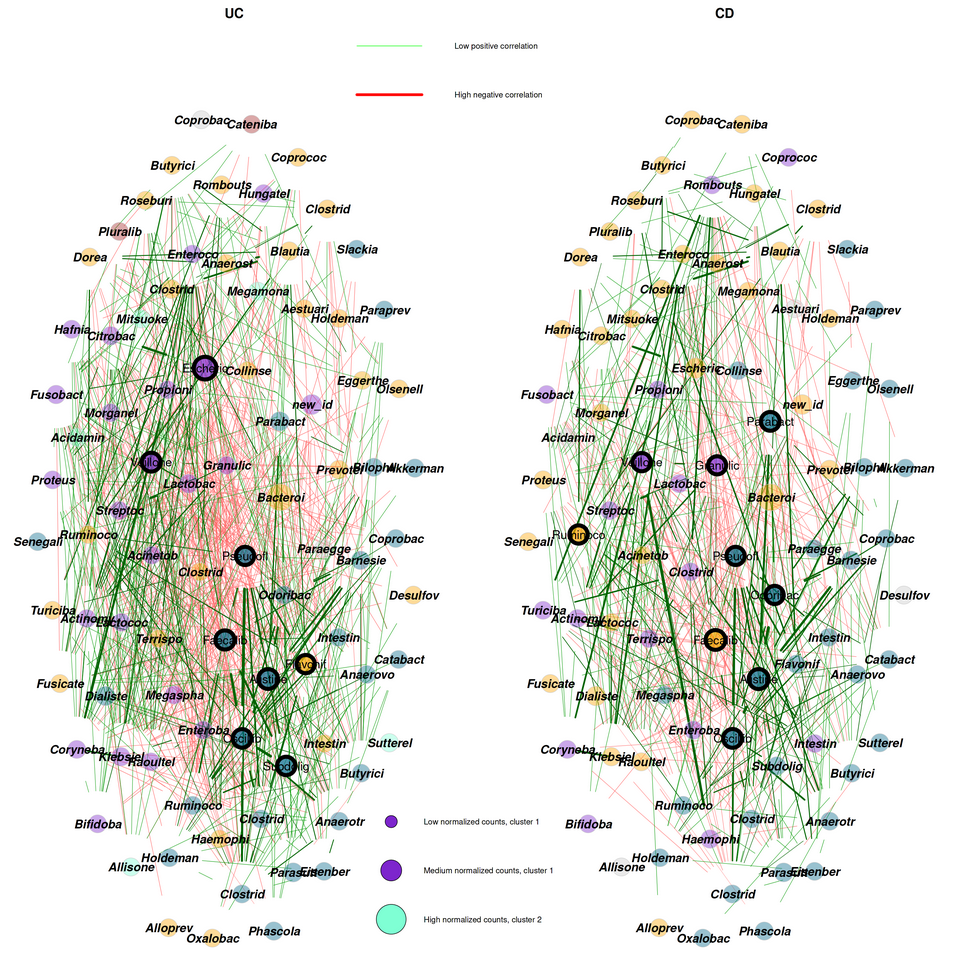
**

Node color depicts the cluster, node size is scaled according to the sum of normalized counts, hubs are highlighted by a black circle, and for clarity only edges corresponding to an absolute association >= 0.15 are plotted and labels of nodes are shortened. The color and thickness of the edges indicate the direction (red for negative, green for positive) and strength of the Pearson correlation coefficient. Same layout is used for both groups (UC and CD) and gray nodes depict genera that are not connected and/or only present in the other group.

**Figure S2**


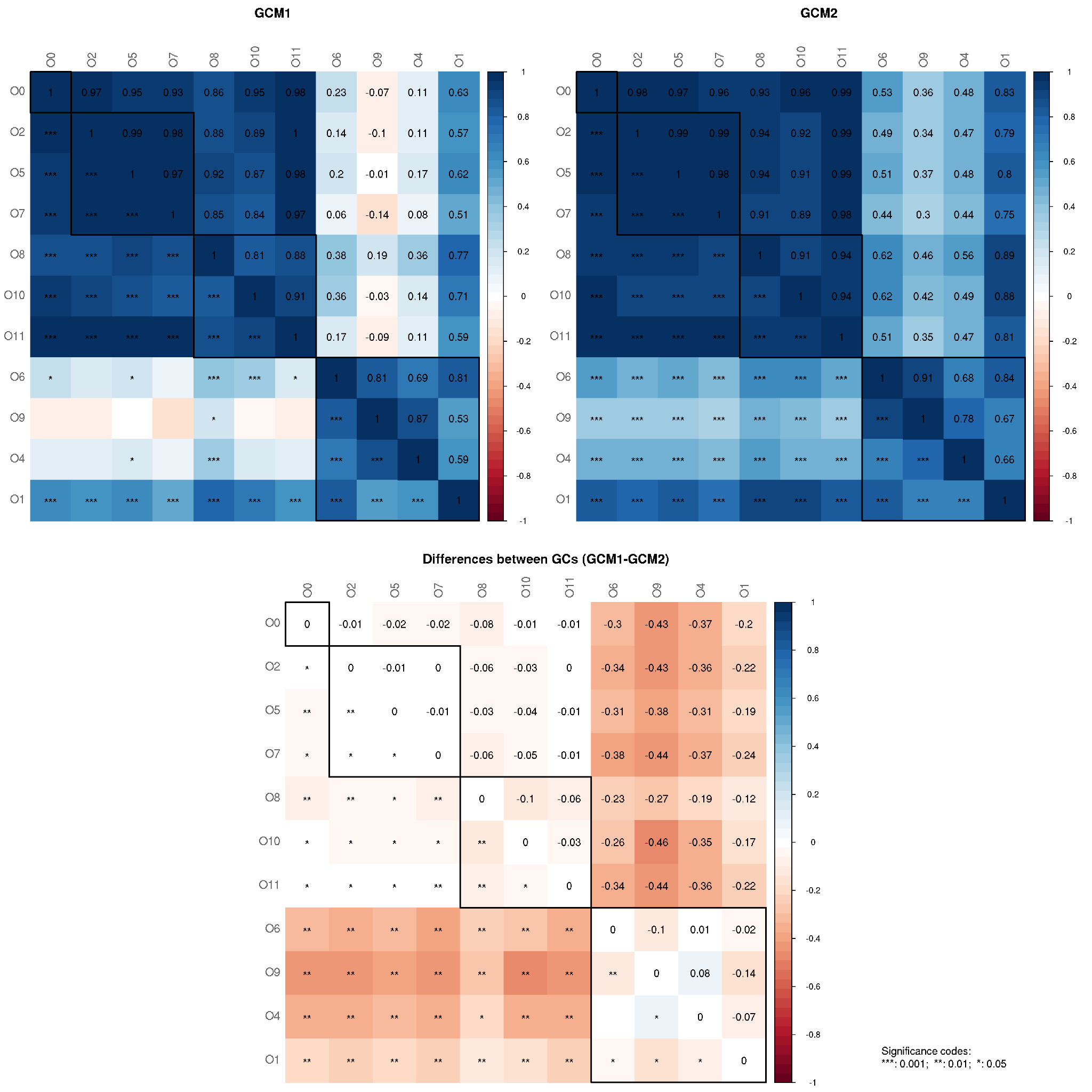


Presented are two graphlet correlation matrices computed for two different networks (based on UC patients (left GCM1) and CD patients (right GCM2)). The graphlet correlation matrix quantifies the pairwise similarity (Spearman correlation coefficient) of subgraph patterns (network's node orbits) in each network. In both cases, the color of each square represents the correlation coefficient between the corresponding pairs of graphlets. The diagonal of the matrices corresponds to the self-correlations of each graphlet. The matrix at the bottom shows the absolute difference between the graphlet correlation matrices for both networks. Positive values (blue) in the difference matrix indicate an increase in similarity, while negative values (red) indicate a decrease. Orbits are sorted according to their topological role.

# References

1. Callahan BJ, McMurdie PJ, Rosen MJ, Han AW, Johnson AJA, Holmes SP. DADA2: High-resolution sample inference from Illumina amplicon data. Nat Methods. 2016;13:581–3.

2. Ruehlemann M. ikmb amplicon processing. 2019.
